# Robust speed control of permanent magnet synchronous motors using model predictive and sliding mode strategies

**DOI:** 10.1371/journal.pone.0343999

**Published:** 2026-03-26

**Authors:** Magdy Meawad, Abdelsalam A. Ahmed, Mona I. Abdelkader, Yasser G. Dessouky

**Affiliations:** 1 Electric and Control Engineering Department, College of Engineering and Technology, Arab Academy, for Science, Technology and Maritime Transport, Alexandria, Egypt; 2 Electrical Power and Machines Engineering Department, Faculty of Engineering, Tanta University, Tanta, Egypt; Qingdao University, CHINA

## Abstract

This paper presents robust speed-control techniques for permanent-magnet synchronous motors (PMSMs) by integrating model-predictive and sliding-mode strategies. Two controllers are developed: a Sliding-Mode Speed Control–based Model Predictive Current Controller (SMSC-MPCC) using an Adaptive Sliding Mode Surface (ASMS) and an Adaptive Improved Exponential Reaching Law (AIERL), and a Model Predictive Direct Speed Controller (MPDSC) incorporating a Sliding-Mode Load Torque Observer (SMLTO) and a Sliding-Mode Disturbance Observer (SMDO) for sensorless operation and delay compensation. The methods are analyzed under explicit disturbance and saturation assumptions, and their stability is established via Lyapunov arguments. Extensive simulations were conducted, encompassing low-speed operation, frequency-sweep tracking, and Monte Carlo–based robustness evaluation under parameter variations. Performance is assessed using rise time, settling time, overshoot, steady-state error, ITAE, chattering index, and closed-loop bandwidth. Simulated tests confirm statistically significant improvements in mean ITAE and stability margin for the proposed SMSC-MPCC across operating conditions, while MPDSC achieves the fastest disturbance recovery with sensorless capability. The results provide a practical pathway to robust, low-chattering speed control under uncertainty, and clarify the trade-offs among predictive sliding-mode designs for PMSM drives.

## 1. Introduction

Permanent magnet synchronous motors (PMSMs) are increasingly favored for applications such as low-power systems, high-performance drives, robotics, electric vehicles, and machine tools. Their growing preference over brush-type motors and gradual replacement of induction motors in many domains can be attributed to superior performance characteristics, including compact design, high air-gap flux density, high power density, high torque-to-inertia ratio, and excellent efficiency [1]. PMSMs are particularly useful in hybrid systems that require flexible propulsion power [2], and their integration with advanced converters improves efficiency in grid-connected renewable energy applications [3]. PMSMs have a tightly coupled, nonlinear, multivariable structure, which makes them sensitive to uncertainties such as parameter changes, external load disturbances, unmodeled dynamics, and nonlinear behaviors [4]. Consequently, many control algorithms have been explored to address these challenges, including field-oriented control (FOC) [5, 6] and Direct Torque Control (DTC) [7]. Pulse- width modulation (PWM) remains the most common traditional method, while advanced approaches such as fuzzy logic [8], Sliding-Mode Control (SMC) [9], and predictive control offer further benefits. In particular, predictive control can deliver superior dynamic performance and speed response compared with conventional methods [10], and computational-intelligence techniques have further improved these methods in renewable energy and in controller parameter auto-tunning [11, 12].

Predictive control can be classified into several categories including: deadbeat, hysteresis, trajectory, and Model Predictive Control (MPC) [13]. MPC uses a system model to predict future behavior of the controlled variables and determine an optimal control action by minimizing a cost function. The cost function directly affects control accuracy and robustness because it can include multiple terms that represent different objectives, such as reference tracking, disturbance rejection, energy minimization, and constraint handling [14, 15].

For PMSM, MPC is typically divided into: finite control set Model Predictive Control (FCS-MPC) and continuous control set Model Predictive Control (CCS-MPC); the need for a modulation unit is a key difference between them [16]. A comparison in reference [17] showed that FCS-MPC delivered comparable performance to CCS-MPC with only a small increase in execution time, and offered higher torque capability at high speed. However, FCS-MPC can increase computational load, which may offset its torque advantages [18]. FCS-MPC has lower design complexity because it uses a limited set of voltage vectors and operates with a variable switching frequency [19], making it the most widely studied and applied predictive technique [20].

This study examines three different FCS-MPC-based control methods for PMSM drives. The goal of these approaches is to improve dynamic response, disturbance rejection, and robustness to parameter mismatch, —key shortcomings of conventional controllers. The baseline is PI-MPCC, which uses an outer-loop PI speed controller to generate the reference quadrature-axis current of the MPCC [21].

The second approach replaces the PI controller with a sliding‑mode speed controller (SMSC) [22, 23]. SMC is known for its robustness, fast transients, and effective disturbance rejection [24]. However, SMC can suffer from chattering due to discontinuous control action [25]. To reduce chattering and improve stability, we adopt an adaptive integral exponential reaching law (AIERL) [26], We further integrate an adaptive sliding surface (ASMS) to improve performance against load and parameter variations [27, 28] this combines adaptation of both the reaching law and the sliding variable [29].

The third technique is a model‑predictive direct speed controller (MPDSC) that uses a sliding‑mode load‑torque observer (SMLTO) to estimate load torque for speed prediction. MPDSC aims to overcome the limitations of cascaded loops in PI‑MPCC and SMSC‑MPCC, which can slow the dynamic response. MPDSC directly controls speed using a cost function designed to improve speed tracking, disturbance reduction, and torque-per-ampere ratio [30, 31].

Accurate load‑torque estimation from the SMLTO enables sensorless operation and increases robustness to disturbances and parameter variations [32–34]. Computational latency is an important challenge for the FCS-MPC and can affect its performance [35]. To mitigate this, we implement delay compensation using a sliding‑mode disturbance observer (SMDO), which enables more accurate one‑step‑ahead current predictions and improves control effectiveness.

The paper is organized as follows: The Methodology and Workflow section describes this study workflow and the used control approaches. The System Mathematical Modelling section develops the mathematical model of the PMSM. The Finite Control Set Model Predictive Control section reviews the baseline controller, PI-MPCC. Proposed Sliding Mode Speed Control-Based Model Predictive Current Control section introduces incorporating the adaptive sliding surface to the SMSC-MPCC. Model Predictive Direct Speed Control with Sliding Mode Load Torque Observer section proposes MPDSC with SMLTO for sensorless operation and delay compensation, using SMDO for accurate current prediction. The Weighting Factors and Control Parameters Tuning section explains how the controllers and observers are tuned using the Simulink response optimizer. Simulation and Results section report the outcomes of the simulation tests. Finally, the Conclusions section summarizes the main findings of the paper.

Key contributions of this study:

1. Dual adaptation in sliding surface and reaching law. We integrate adaptation into both the sliding surface and the reaching law. The results indicate that adapting both components yield optimal performance in terms of transient response and THD% robustness under parameter mismatch.
2. Integration of SMDO-based delay compensation and adaptive SMLTO in MPDSC. We incorporate sliding mode disturbance observer for two-step delay compensation in the MPDSC, and we add adaption to the reaching law within the SMLTO. The results show that these additions improve MPDSC robustness and reducing the impact of parameter mismatch on system performance.

## 2. Methodology and workflow

**Fig 1:**
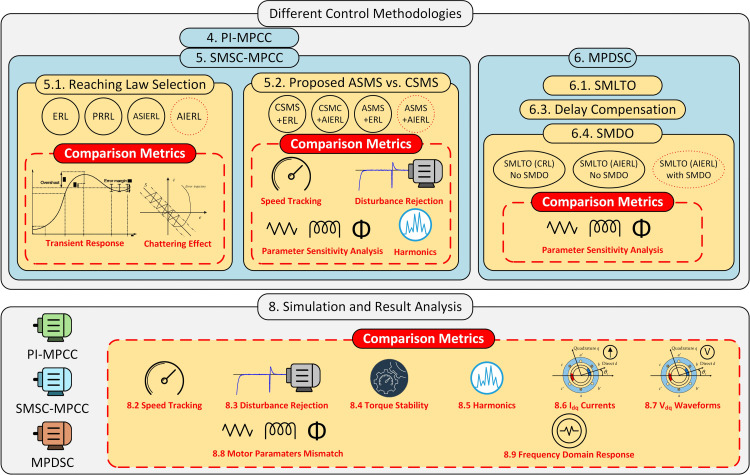
Workflow of the proposed study comparing PI-MPCC, SMSC-MPCC, and MPDSC controllers.

This study follows the workflow shown in Fig 1 to compare the three control approaches, PI-MPCC (baseline), SMSC-MPCC and MPDSC and to identify the most robust method under internal and external disturbances. The comparison procedure is as follows:

1. PI-MPCC (Section 4).
2. SMSC-MPCC (Section 5) with two design studies:
  a. Reaching-law selection (Section 5.1): ERL, PRRL, ASIERL, and AIERL
  b. proposed combination of ASMS + AIERL (Section 5.2), benchmarked against CSMS using both conventional and adaptive reaching laws.
3. MPDSC (Section 6), which includes:
  a. An SMLTO for load-torque estimation with adaptive reaching law.
  b. SMDO-based delay compensation for current-variation estimation.
  c. For comparison with recent literature, we also consider (a) SMLTO with a conventional reaching law and (b) a delay-compensation variant that uses traditional current prediction (no SMDO).

Each block in Fig 1 lists the metrics used for design decisions and controller comparison, including: transient response in the time domain, Disturbance-Rejection capability, parameter-sensitivity analysis, and harmonic content. Section 8 presents a system-level comparison for each scenario and controller. Time-domain and frequency-domain results are logged, and dispersion statistics are compiled to support the conclusions in Section 9.

## 3. System mathematical modelling

### 3.1. Continuous state space modeling of PMSM

The stator voltage equation of PMSM is shown in (1).


$$v_{x}(t)=R_{s}i_{x}(t)+\frac{d}{dt}\lambda_{x}(t)=\;R_{s}i_{x}(t)+L_{s}\frac{d}{dt}i_{x}(t)+e_{x}(t)$$ (1)

where $v_{x}$ is the phase (a, b, or c) voltage, $R_{s}$ is the stator resistance, $i_{x}$ is the phase current, $L_{s}$ is the stator inductance, $\lambda_{x}$ is the phase flux linkage, $e_{x}$ is the phase back electromotive force.

Eq. (1) shows that the flux linkage depends not only on the current in each phase, but also on the mutual flux produced by currents in the other phases and on the rotor speed $\left(\omega_{r}\right)$ (where $e_{x}=f(\omega_{r})$). The complexity of these equations and the flux-linkage computations can be reduced by changing the reference frame. By applying Clarke’s transformation, the three-phase stationary a-b-c system is represented as a two-dimensional space vector. This vector can then be converted to the rotating dq0 reference frame that turns at the rotor speed. As a result, the sinusoidal ABCs components become two DC components in the dq frame, which is easier to regulate. The mathematical model of the Permanent Magnet Synchronous Motor (PMSM) in the d-q coordinate system is given as follows [36],


$$v_{d}(t)=\;i_{d}(t)\;R_{s}+\;L_{s}\frac{d}{dt\;}i_{d}(t)-\;{L_{s}\omega }_{e}(t)\;i_{q}(t)$$ (2)

$$v_{q}(t)=\;i_{q}(t)\;R_{s}+L_{s}\frac{di_{q}(t)}{dt\;}+L_{s}\omega_{e}(t)i_{d}(t)+\omega_{e}(t)\lambda_{pm}$$ (3)

Hence, the differential equations of stator currents are shown below:


$$\frac{d}{dt\;}i_{d}(t)=\;\frac{\text{1}}{L_{s}}\left(-\;i_{d}(t)\;R_{s}+v_{d}(t)+\;L_{s}\;\omega_{e}(t){\;i}_{q}(t)\right)$$ (4)

$$\frac{d}{dt\;}i_{q}(t)=\frac{\text{1}}{L_{s}}\left({-i}_{q}(t)R_{s}+v_{q}(t)-L_{s}\omega_{e}(t)\;i_{d}(t)-\omega_{e}(t)\lambda_{pm}\right)$$ (5)

where, $i_{d}$ and $i_{q}$ are stator current in d- and q-axis respectively; $v_{d}$ and $v_{q}$ are the stator voltages in d- and q-axis respectively; $\lambda_{pm}$ is the permanent-magnet flux linkage; and $\omega_{e}$ is the electrical rotor speed. *L*s denotes the stator inductance in the d- or q-axis (we assume a non-salient PMSM). For the electro-mechanical model, the electromagnetic torque $T_{e}$ can be expressed as $T_{e}=\frac{\text{3}}{\text{2}}p\;\left(\lambda_{d}i_{q}-\lambda_{q}i_{d}\right).$ With $\lambda_{d}=L_{d}i_{d}+\lambda_{PM}$, $\lambda_{q}=L_{q}i_{q}$, this becomes $T_{e}=\frac{\text{3}}{\text{2}}p\left(\lambda_{PM}i_{q}+\left(L_{d}-L_{q}\right)i_{d}i_{q}\right).$ For a non-salient PMSM where $L_{d}=L_{q}$, the reluctance term vanishes, yielding


$$T_{e}(t)=\frac{\text{3}}{\text{2}}p\lambda_{pm}\;i_{q}(t).$$ (6)

The mechanical dynamics of the rotor are then described by


$$\frac{d\omega_{m}(t)}{dt}=\frac{\text{1}}{J_{eq}}\;\left(T_{e}(t)-B_{m}\omega_{m}(t)-T_{L}(t)\right).$$ (7)

where $\omega_{m}$ is the mechanical rotor speed and p denotes the number of pole pairs, so $\omega_{m}=\omega_{e}/p\;$. $J_{eq}$ is the equivalent moment of inertia of the machine and load, $B_{m}$ is the viscous friction coefficient, and $T_{L}$ is the load torque.

### 3.2. Discretization of PMSM model

Applying the Euler’s forward discretization to equations (4), (5), (6) and (7), we obtain the predicted expressions for the current components as follows:


$$\overset{p}{\underset{d}{i}}(k+\text{1})=i_{d}(k)+T_{s}\left(\frac{\text{1}}{L_{s}}\left(-i_{d}(k)R_{s}+v_{d}(k)+\;\omega_{e}(k)L_{s}i_{q}(k)\right)\right)$$ (8)

$$\overset{p}{\underset{q}{i}}(k+\text{1})=i_{q}(k)+\frac{T_{s}}{L_{s}}\left({-\;i}_{q}(k)R_{s}+v_{q}(k)-\omega_{e}(k)L_{s}i_{d}(k)-\omega_{e}{(k)\lambda }_{pm}\right)$$ (9)

$$\overset{p}{\underset{e}{\omega }}(k+\text{1})=\;\omega_{e}(k)+\frac{T_{s}}{J_{eq}}\left(\frac{\overset{p}{\underset{e}{T_{e}(k)+T}}(k+\text{1})}{\text{2}}-B_{m}\omega_{e}(k)-T_{L}(k)\right)$$ (10)

where $\overset{p}{\underset{d,q}{i}}(k+\text{1})$ are the predicted dq axes currents at instant (k+1), and $\overset{p}{\underset{e}{\omega }}(k+\text{1})\;$ is the predicted electrical speed at (k+1). Thus, a discretized model is obtained, which can be used in Model predictive control [37].

### 3.3. Inverter model

**Fig 2:**
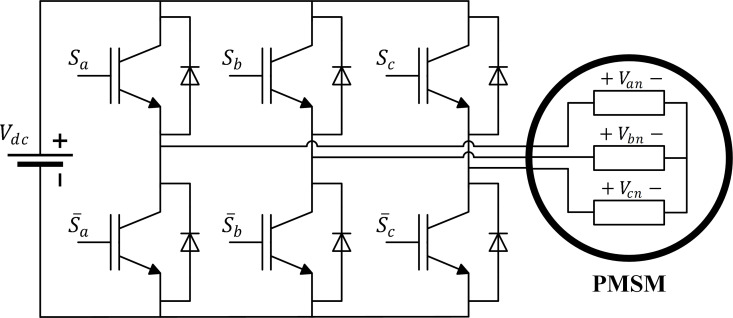
Two-level three-phase voltage source inverter.


The two-level full-bridge Voltage Source Inverter (VSI) shown in Fig 2 is used in the simulations; each IGBT is modeled as an ideal switch. The terminal voltage in the dq-reference frame for the Y-connected PMSM are line-to-neutral voltages that are required and can be obtained as described below:

$$\begin{array}{c} \left[\begin{array}{c} V_{an}\\ V_{bn}\\ V_{cn} \end{array}]=\frac{V_{dc}}{\text{3}}\;[\begin{array}{ccc} \text{2} & -\text{1} & -\text{1}\\ -\text{1} & \text{2} & -\text{1}\\ -\text{1} & -\text{1} & \text{2} \end{array}]\;[\begin{array}{c} S_{a}\\ S_{b}\\ S_{c} \end{array}\right] \end{array}$$ (11)

where S is the voltage vector that depends on the switching function: it equals 1 when the upper-leg IGBT is ON and 0 when it is OFF.

The inverter switching voltages can be obtained directly in the dq-reference frame as in (12) [38],


$$\begin{array}{c} \left[\begin{array}{c} v_{d}\\ v_{q} \end{array}]=\frac{{\text{2}V}_{dc}}{\text{3}}\;[\begin{array}{ccc} \text{cos}(\theta ) & \text{cos}\left(\theta -\frac{\text{2}\pi }{\text{3}}\right) & \text{cos}\left(\theta +\frac{\text{2}\pi }{\text{3}}\right)\\ -\text{sin}(\theta ) & -\text{sin}\left(\theta -\frac{\text{2}\pi }{\text{3}}\right) & -\text{sin}\left(\theta +\frac{\text{2}\pi }{\text{3}}\right) \end{array}][\begin{array}{c} S_{a}\\ S_{b}\\ S_{c} \end{array}\right] \end{array}$$ (12)

## 4. Finite control set model predictive control

As noted above, the PI-MPCC is implemented as the baseline for comparison with the other control approaches. Its main components and structure are similar to the SMSC-MPCC block diagram shown in Fig 5, except that the outer-loop Controller is a PI controller instead of the SMSC. The PI controller maintains the motor speed at the desired reference. The inverter switching state is selected by minimizing a cost function that reduces the error between the predictive quadrature current, $\overset{p}{\underset{q}{i}},$ and the reference quadrature current, $\overset{*}{\underset{q}{i}}$, thereby achieving the desired reference speed. A straightforward cost function for this objective is:

**Fig 3:**
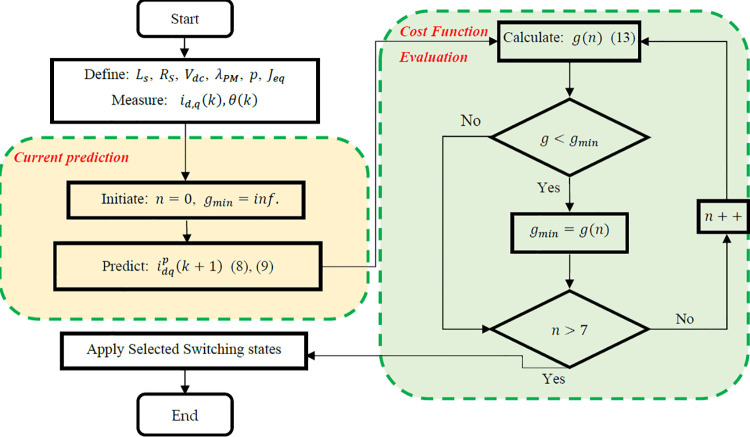
Flowchart of the PI-based model predictive current controller (PI-MPCC).

**Fig 4:**
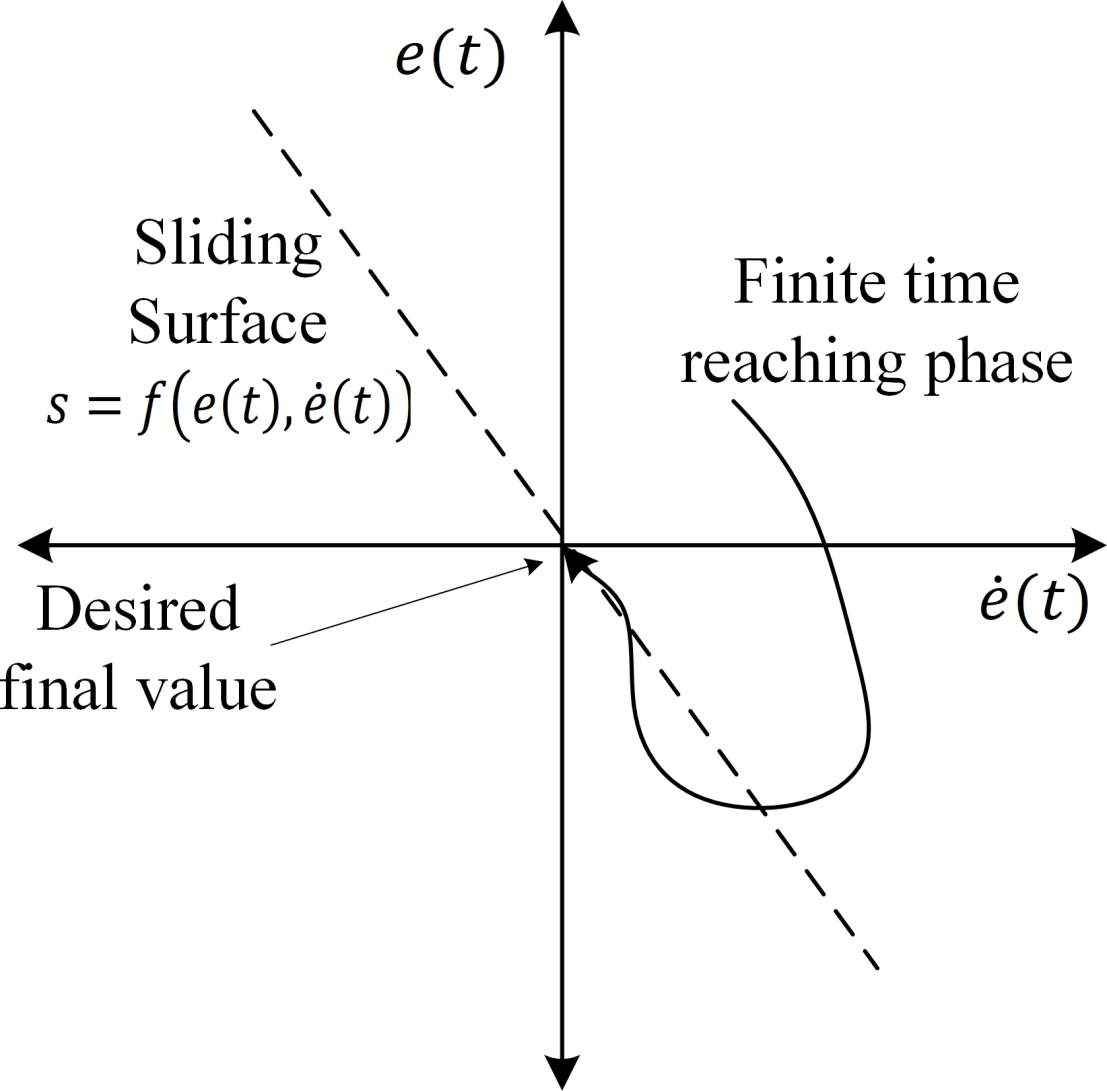
Graphical interpretation of sliding-mode control showing the system trajectory reaching and sliding along the sliding surface.


$$g={k_{d}\;\left(\overset{p}{\underset{d}{i}}(k+\text{1})\right)}^{\text{2}}+{k_{q}\left(\overset{*}{\underset{q}{i}}-\overset{p}{\underset{q}{i}}(k+\text{1})\right)}^{\text{2}}$$ (13)

where $k_{d}$ and $k_{q}$ are weighting factors for the d-axis and the q-axis currents, respectively. The first term reduces reactive power; the d-axis current reference, $\overset{*}{\underset{d}{i}}$, is set to zero to optimize torque per ampere [39]. The second term monitors the torque -producing current. The cost function [13] evaluates the discrepancy between predicted and reference currents and identifies the voltage vector that minimizes this error. Fig 3 presents a flowchart of these steps, where n represents the number of possible switching states, constrained to 8 iterations corresponding to the potential voltage vectors. In each iteration, the predicated values of $\overset{p}{\underset{d}{i}}(k+\text{1})$ and $\overset{p}{\underset{q}{i}}(k+\text{1})$ are computed for each switching state $S_{\text{1}\to \text{6}}$.

The predicted currents are compared to the reference currents$\;\overset{*}{\underset{q}{i}}$ and $\overset{*}{\underset{d}{i}}$ using the chosen cost function. The optimal voltage vector is selected and applied directly to the inverter by setting the corresponding leg switch states. The currents are measured at sampling time (k), and the MPCC predicts the eight possible current vectors at the next sampling instant (k+1). These vectors comprise six active vectors $\left(v_{\text{1}}-v_{\text{6}}\right)$ and two zero vector voltages $\left(v_{\text{0}},v_{\text{7}}\right)$. The eight vectors are produced by different switch-states combinations $\left(S_{a},S_{b},S_{c}\right)$ as shown in Table 1 [40].

**Table 1:** Voltage vectors of the two-level inverter corresponding to different switching states.

State	${𝐒}_{a}$	${𝐒}_{b}$	${𝐒}_{c}$	${𝐕}_{an}$	${𝐕}_{bn}$	${𝐕}_{cn}$	Voltage Vector
0	0	0	0	0	0	0	*v*_*0*_ = 0∠0
1	1	0	0	2/3 V_dc_	−1/3 V_dc_	−1/3 V_dc_	*v*_*1*_ = 2/3 *V*_*dc*_*∠0*
2	1	1	0	1/3 V_dc_	1/3 V_dc_	−2/3 V_dc_	*v*_*2*_ = 2/3 *V*_*dc*_*∠60*
3	0	1	0	−1/3 V_dc_	2/3 V_dc_	−1/3 V_dc_	*v*_*3*_ = 2/3 *V*_*dc*_*∠120*
4	0	1	1	−2/3 V_dc_	1/3 V_dc_	1/3 V_dc_	*v*_*4*_ = 2/3 *V*_*dc*_∠180
5	0	0	1	−1/3 V_dc_	−1/3 V_dc_	2/3 V_dc_	*v*_*5*_ = 2/3 *V*_*dc*_∠240
6	1	0	1	1/3 V_dc_	−2/3 V_dc_	1/3 V_dc_	*v*_*6*_ = 2/3 *V*_*dc*_∠300
7	1	1	1	0	0	0	*v*_*7*_ = 0∠0

## 5. proposed sliding mode speed control-based model predictive current control SMSC-MPCC

This section introduces the SMSC-MPCC method, designed to improve control accuracy and robustness in PMSM systems using sliding-mode principles. SMC is based on defining a scalar function of the system states, called the sliding surface. The control objective is to drive the system’s trajectory to this surface when an error occurs and then maintain sliding along the surface so the tracking error converges to zero, even in the presence of parameter uncertainties or external disturbances. The control law is designed to guarantee this behavior, as shown in Fig 4.

**Fig 5:**
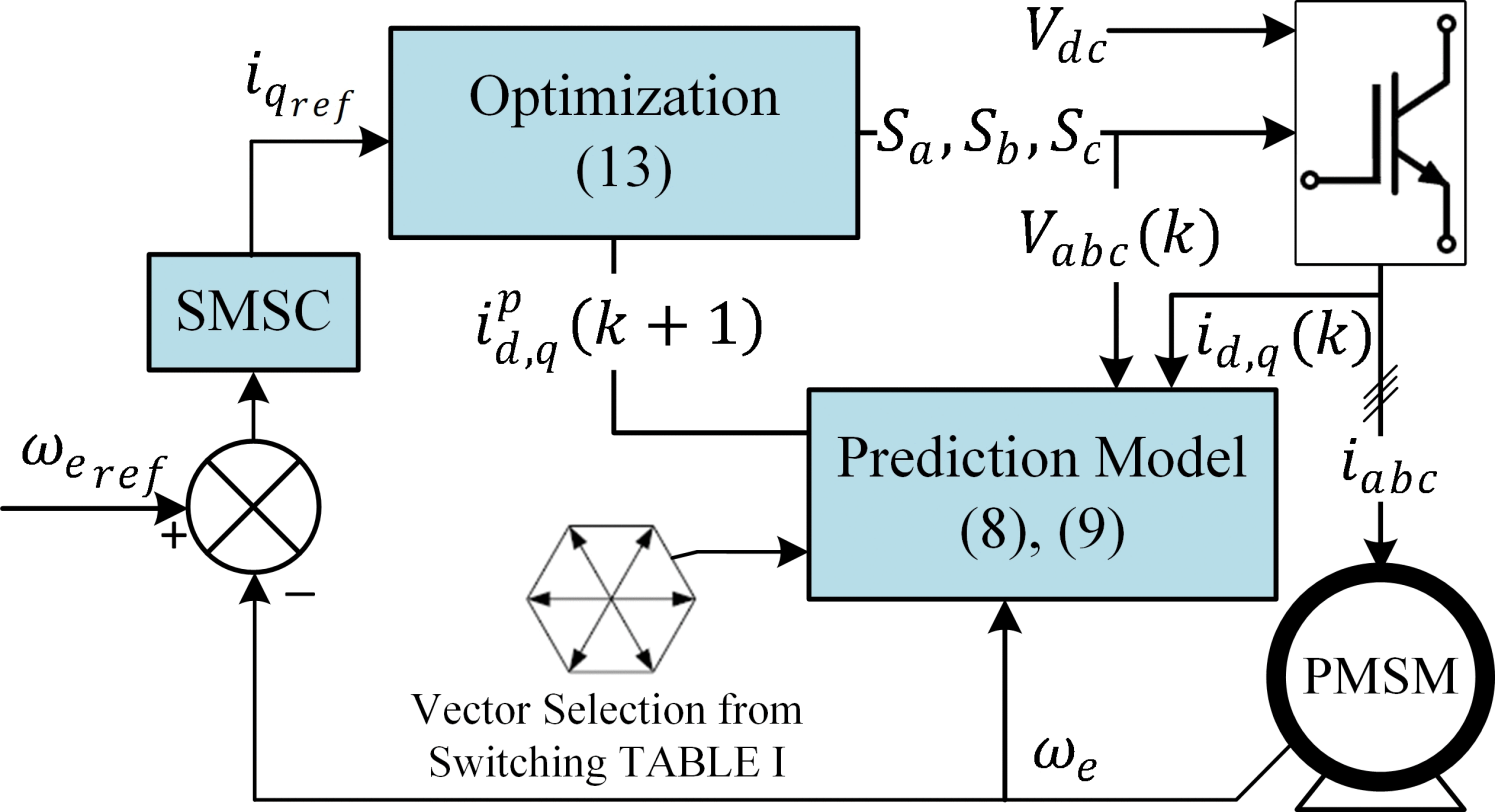
Block diagram of the proposed sliding-mode speed control-based model predictive current controller (SMSC-MPCC).

The SMSC-MPCC scheme is shown in Fig 5. The outer-loop controller, SMSC, is employed to precisely control the speed to match the desired reference. This approach uses the same cost function given in [13]. The speed-tracking error is expressed as follows:


$$e(t)=\omega_{ref}(t)-\omega_{e}(t),$$ (14)

Where $\omega_{ref}(t)$ denotes the reference speed.

SMC design consists of two key phases:

1. Select the sliding surface.
2. Design a control law that drives the trajectory onto the sliding surface (reaching phase) and maintains it there until the error converges to zero (sliding phase). The reaching-phase condition is often framed via a candidate Lyapunov function such as [15] to ensure stability in the Lyapunov sense:

$$V=\frac{\text{1}}{\text{2}}s^{\text{2}}\;$$ (15)

Where s is the sliding variable; as long as $\dot{V}<\text{0}$ for s≠0. Zero convergence assured according to [16].


$$s\;\cdot \dot{s}<\text{0}\;$$ (16)

The sliding surface is defined in [41], for $n^{th}$ order, sliding surface is given by


$$s(t)={\left(\frac{d}{dt}+c\right)}^{n-\text{1}}e(t).$$ (17)

Then, the conventional sliding surface for the 2^nd^ order (n-1) nonlinear model can be expressed as follows:


$$s(t)=\dot{e}(t)+c\;\cdot e(t)\;$$ (18)

where c > 0 is the sliding mode gain or the sliding surface slope.

With the sliding surface definition, as s approaches zero, then we obtain e ˙=−ce which implies that e(t)→0 exponentially at t→ ∞. The reaching law must be formulated to satisfy the sliding condition given in [16], guaranteeing that Lyapunov derivative remains negative and directing the system toward the sliding surface. Accordingly, the reaching law for achieving constant-velocity is defined as follows:


$$\;\dot{s}=-\eta \;sgn(s),\;\eta >\text{0}\;$$ (19)

### 5.1. Reaching law selection

The design of reaching law is a crucial step in SMC, since it dictates how the system state trajectory reaches the sliding surface. The specific formulation of the reaching law strongly affects controller performance and robustness. In this study, several commonly used reaching laws are examined and compared to identify the most effective law for the proposed control approaches. Comparison metrics include convergence rate, chattering suppression, and disturbance rejection capability.

The constant-velocity reaching law, as outlined in [19], drives s toward the sliding manifolds at a constant rate. Its advantages are simplicity and ease of implementation. However, it tends to induce significant chattering, so alternative strategies are considered to mitigate this effect. One such strategy is the Exponential Reaching Law (ERL), which introduces a continuous proportional term to reduce the abrupt control action [42]:


$$\dot{s}=-\eta \;sgn(s)-K\;s,\;K>\text{0}.\;$$ (20)

By adding the proportional term (−K s), ERL improves convergence speed and reduces chattering. Excessive suppression of chattering, however, can slow the system response—a trade-off that motivated more advanced reaching laws. A further enhancement is the power-rate reaching law (PRRL), designed to accelerate convergence when s is too large [43]:


$$\dot{s}=\;-\;\eta \;|s{|}^{a}\;sgn(s),\;\text{0}<a<\text{1}.$$ (21)

PRRL achieves rapid initial convergence but doesn’t inherently limit control effort. Without external constraints, this may produce excessively large control actions that can degrade performance or cause instability.

To combine the benefits of ERL and PRRL while mitigating their drawbacks, an Improved Exponential Reaching Law (IERL) was proposed in [26]:


$$\dot{s}=-\eta \;|x{|}^{a}\;sgn(s)-K\;s,\;\text{0}<a<\text{1}$$ (22)

where in this formulation x represents a state variable or sliding variable. This combination balances reaching time and chattering by dynamically adjusting control effort with the tuning parameters (η, a, K), which significantly influence convergence time and control smoothness.

An additional refinement, the Augmented-State Improved Exponential Reaching Law (ASIERL) [44], incorporates multiple state and sliding variables into the reaching law as follows:


$$\dot{s}=-\left(m_{\text{1}}\;{\left|x_{\text{1}}\right|}^{a}+m_{\text{2}}\;{\left|x_{\text{2}}\right|}^{b}+\;m_{\text{3}}\;|s{|}^{c}\right)\;sgn(s)-K\;s\;$$ (23)

where $\;\underset{t\to \infty }{\text{lim}}\left|x_{\text{1}}\right|=\text{0},\;\underset{t\to \infty }{\text{lim}}\left|x_{\text{2}}\right|=\text{0}\;\underset{t\to \infty }{\text{lim}}|s|=\text{0},\;{\;x}_{\text{1}}=e(t),\;x_{\text{2}}=\dot{x_{\text{1}}}=\dot{e}(t),\;m_{\text{1}}>\text{0},\;m_{\text{2}}>\text{0}\text{ and }m_{\text{3}}>\text{0}$ and a,b,c ∈(0,1).  In ASIERL, each term vanishes as t→∞, ensuring asymptotic convergence. By using the system state and its derivatives, ASIREL improves disturbance rejection and smooths the control action.

Building upon these ideas, reference [26] proposed an adaptive gain version of IERL, termed the Adaptive Improved Exponential Reaching Law (AIERL) as specified in (24, 25).


$$\dot{s}=-\frac{\eta }{F(s)}\;|s{|}^{a}\;sgn(s)-K\;s\;$$ (24)

$$F(s)=m+(\text{1}-m)e^{-b|\;s|}.\;\text{0}<a,m<\text{1}$$ (25)

**Fig 6:**
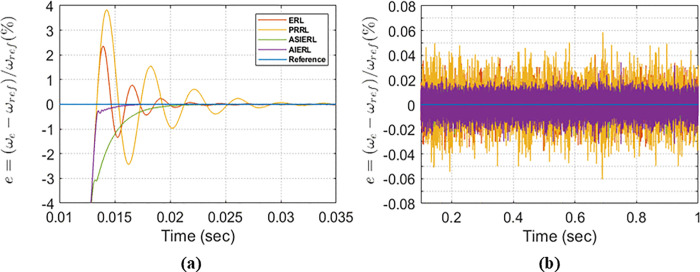
Comparison of reaching-law performance: (a) transient system response; (b) steady-state chattering level.

Here m and b are tuning parameters that control adaptation strength, and adaptation rate, respectively. AIERL combines the advantages’ features of PRRL and ERL by providing an extra degree of freedom to trade off chattering magnitude versus reaching time. Fig 6 presents a comparative analysis of these reaching laws in terms of transient convergence and chattering. The results indicate that, during the transient phase, PRRL and IERL exhibit larger overshoots than AIERL. ASIERL and AIERL both effectively reduce chattering as illustrated in Fig 6(b) but AIERL achieves the shortest settling time as shown in Fig 6(a). Consequently, AIERL is adopted in both the SMSC-MPCC and MPDSC controllers.

### 5.2. Proposed Adaptive Sliding Mode Surface (ASMS)

An adaptive reaching law is proposed in [45, 46]. In this paper, we propose adapting the sliding surface for the same purpose: to provide speed-control quality and disturbance rejection. ASMS changes the slope of sliding surface according to the speed error, forcing the surface towards the error value and accelerating the convergence to zero error [47].

Eq. (17) for CSMS shows that control performance is governed by a constant sliding-mode gain, which is independent of motor characteristics and external disturbances. Proper tuning of c is therefore essential for resilience and dynamic response. Higher values of c improve the controller’s ability to reject unknown disturbances but increase sensitivity to the error and its derivative, producing significant chattering. Lower values of c reduce chattering but can weaken disturbance rejection in steady state and may degrade robustness. Consequently, the conventional sliding‑mode controller often struggles to achieve both fast transient performance and strong steady‑state disturbance rejection. To mitigate this trade-off, this paper presents an adaptive sliding mode surface as shown in (29).


$$s_{A}(t)=\dot{e}(t)+f_{c}(e)\;\cdot e(t)\;$$ (26)

**Fig 7:**
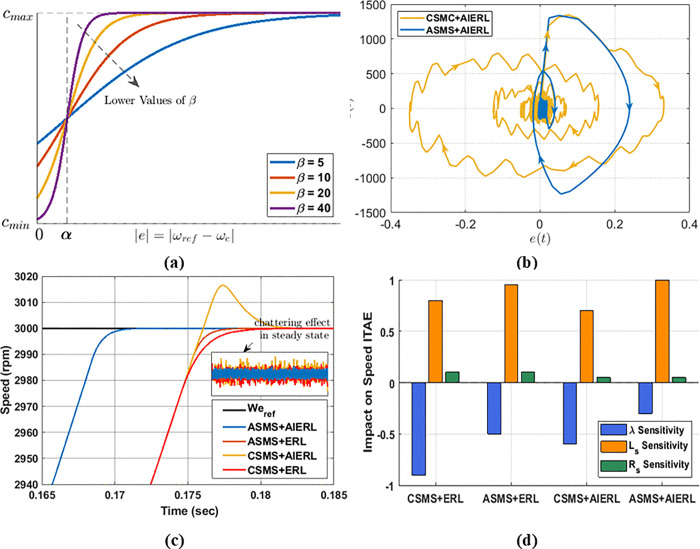
Comparison of different SMSC approaches combining adaptive sliding-surface and reaching-law designs: (a) adaptive function ${𝐟}_{c}(e)$; (b) phase trajectories; (c) transient and chattering responses; (d) parameter-sensitivity analysis.

The adaptation uses a gain function $f_{c}(e)$, a sigmoid-shaped function of the absolute error [48], which provides a smooth transition between $c_{min}$ and $c_{max}$, and avoids abrupt changes in the control action. This sigmoid function in [27] is defined as follows:


$$f_{c}(e)=\;c_{min}+\frac{c_{max}-c_{min}\;}{\text{1}+e^{-\beta (|e|-\alpha )}}\;$$ (27)

where, $c_{min}$ and $c_{max}$ bound the sliding gain, α sets the error bandwidth over which c, β controls the steepness of the transition as shown in Fig 7(a). For large error, c is set near $c_{max}$ to prioritize fast convergence; for small errors, c is reduced toward $c_{min}$ to optimize steady-state performance and disturbance rejection. In this way, the adaptive sliding surface $s_{A}$ enables the controller to adapt to changes in reference speed and to external disturbances.

To assess the impact of combining effect of an adaptive sliding surface and adaptive reaching law, we implemented and compared four control strategies:

1. CSMS with conventional RL such as ERL.
2. ASMS with ERL [49].
3. CSMS with an adaptive RL such as AIERL [50].
4. The proposed approach: ASMS combined with AIERL.

Fig 7(b) shows phase trajectory comparison demonstrating disturbance-rejection capability of CSMS and ASMS in response to a step change in load torque (from rated torque to no-load). The sliding-surface adaptation reduces speed deviation by approximately 40%. Fig 7(c) presents transient response, confirming that ASMS+AIERL achieves the shortest settling time and the lowest chattering ripples among the tested methods. Combining ASMS with an adaptive RL such as AIERL yields significant improvements in robustness to parameter mismatch disturbances, chattering suppression, and THD% compared with using sliding-surface adaption or adaptive reaching law alone.

A parameter-sensitivity analysis, as shown in Fig 7(d), was performed for all scenarios. The results indicate that the ASMS +AIERL combination provides the most robust and balanced response, especially against variations in $L_{s}$ and $\lambda_{PM}$. In contrast, the conventional sliding surface with either ERL or an adaptive RL show stronger dependence on system parameters as illustrated in Fig 7(d).

Table 2 further summarizes the sensitivity and harmonic-distortion results, demonstrating that the proposed ASMS + AIERL configuration achieves the lowest THD% and the highest positive impact on parameter robustness, confirming its superior overall performance among the tested strategies.

**Table 2:** Performance comparison of conventional and adaptive sliding-mode combinations for sliding-variable and reaching-law design, including parameter sensitivities, THD%, and qualitative trade-offs.

Control Strategy	${𝐑}_{s}$ Sensitivity	${𝐋}_{s}$ Sensitivity	$\lambda_{PM}$ Sensitivity	THD %	Advantages	Limitations
CSMS + ERL	Negligible	High Positive Impact	Highest Negative Impact	6.69	Inherent robustness to bounded uncertainties Simpler implementation	Trade-off between fast reaching and chattering. Slower response in disturbances.
ASMS + ERL [49]	Negligible	High Positive impact	Moderate Negative Impact	5.42	Improved dynamic stability and response speed	Moderate overall robustness and chattering mitigation.
CSMS + AIERL [50]	Negligible	Moderate Positive Impact	High Negative Impact	6.68	Faster reaching with minimal overshoot. Enhanced disturbance rejection.	Less effective for parameter mismatch Require bounds on uncertainties
ASMS + AIERL (This paper)	Negligible	Highest Positive Impact	Lowest Negative Impact	4.82	Fastest convergence Lowest chattering Superior overall robustness.	More complex and computational demand.

### 5.3. Sliding Mode Speed Control (SMSC) Design

The adaptive sliding surface $s_{A}$ was defined in [26]. Its time derivative is obtained in [28]:


$${\dot{s}}_{A}=\;\ddot{e}+\;f_{c}(e)\cdot \dot{e}+\;{\dot{f}}_{c}(e)\cdot e$$ (28)

where $\dot{e}$ is the rate of change of the speed-tracking error and it is numerically approximated using a backward-difference as shown in [29]. Substituting equation (30) into [28] gives [31]:


$$\dot{e}\approx \frac{e(k)-e(k-\text{1})}{T_{s}}$$ (29)

$${\dot{f}}_{c}(e)=\frac{\left(c_{max}-c_{min}\right)\;e^{-\beta (|e|-\alpha )}\beta \;sgn(e)\;\dot{e}}{{\left(\text{1}+e^{-\beta \left(|e|-\alpha \right)}\right)}^{\text{2}}}$$ (30)

$${\dot{s}}_{A}=\;\ddot{e}+\;f_{c}(e)\cdot \dot{e}\;+\;\left(\frac{\left(c_{max}-c_{min}\right)\;e^{-\beta (|e|-\alpha )}\beta \;sgn(e)\;\dot{e}}{{\left(\text{1}+e^{-\beta \left(|e|-\alpha \right)}\right)}^{\text{2}}}\right)\cdot e$$ (31)

Assuming $\omega_{ref},\;T_{L}$ are constants (i.e., their derivatives are zero) and neglecting $B_{m}$ and then, using [14] together with substitution from [7], the derivative of the sliding surface becomes [32].


$${\dot{s}}_{A}=-{\ddot{\omega }}_{e}\;+\;f_{c}(e)\cdot \dot{e}+\;{\dot{f}}_{c}(e)\cdot e=-\frac{\text{3}\;p^{\text{2}}\;\lambda_{PM}\;}{\text{2}\;J_{eq}}\;{\dot{i}}_{q}(t)+f_{c}(e)\cdot \dot{e}+\;{\dot{f}}_{c}(e)\cdot e$$ (32)

**Fig 8:**
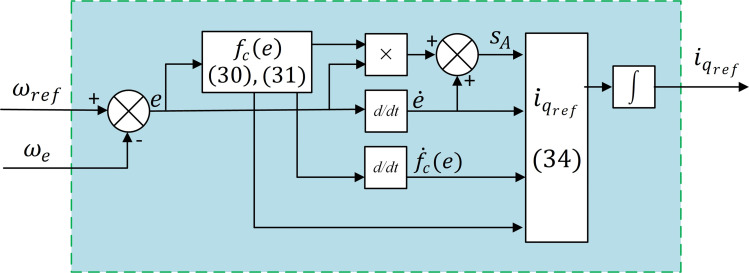
Block diagram of the adaptive sliding-mode speed controller implementing ASMS and AIERL for speed regulation.

To enforce convergence of $s_{A}$ to zero, we apply AIERL in [24]. Integrating both sides of [32] with the Forward-Euler numerical method produces the desired reference for the q-axis current, as shown in [33]. The schematic of this SMSC implementation is illustrated in Fig 8.


$$i_{q_{ref}}=\frac{\text{2}J}{\text{3}p^{\text{2}}\lambda_{PM}}\int f_{c}(e)\cdot \dot{e}+\;{\dot{f}}_{c}(e)\cdot e+\frac{\eta }{F\left(s_{A}\right)}{\left|s_{A}\right|}^{a}\;sgn\left(s_{A}\right)+Ks_{A}dt$$ (33)

### 5.4. Stability analysis

Theorem 1 (Global Asymptotic Stability of the Proposed SMSC-MPCC):

Consider the PMSM system controlled by the proposed SMSC-MPCC, employing the ASMS defined in [26] and the AIERL defined in [24].

Assume that the adaptive sliding gain c(e) is strictly positive, bounded, and continuously differentiable, such that $\text{0}<c_{min}\leq c(e)\leq c_{max}$ and that its time derivative $\dot{c}(e)$ is bounded and AIERL parameters satisfy K,η, b>0 and 0<a,m<1 with $F(s_{A})\geq \text{0}$.

Proof:

Define the Lyapunov candidate function given in [15], with $V_{s}(\text{0})=\text{0}$ and $V_{s}(t)>\text{0}$ for s(t)≠0. The transition of the error trajectory from the reaching phase to the sliding phase depends on the chosen control strategy; this requirement is commonly called the reaching condition. A sufficient condition to guarantee this reaching condition is shown in [34]:


$$\dot{V_{s}}=s_{A}\;\dot{s_{A}}<\text{0},\;s_{A}\neq \text{0}.\;$$ (34)

Substituting the reaching law yields


$$\dot{V_{s}}=s_{A}\left(-\frac{\eta }{F(s)}\;{\left|s_{A}\right|}^{a}\;sgn\left(s_{A}\right)-K\;s_{A}\;\right)=-\frac{\eta }{F\left(s_{A}\right)}s_{A}{\left|s_{A}\right|}^{a+\text{1}}-K\overset{\text{2}}{\underset{A}{s}}\;$$ (35)

With the parameter constraints, K,η, b>0 and 0<a,m<1, stability is ensured if $\dot{V_{s}}<\text{0}$ for all $s_{A}\neq \text{0}$. This condition implies that the system trajectories converge asymptotically to the adaptive sliding surface s=0. Ensuring e→0 exponentially. This guarantees robustness to parameter variations and disturbances, as validated by simulations showing reduced ITAE and THD% as declared in Table 2.

In conclusion, the proposed SMSC with ASMS and AIERL guarantees global asymptotic stability. The following section represents the MPDSC approach as a benchmark to further evaluate SMSC-MPCC performance. This comparison assesses how omitting the outer‑loop SMSC affects robustness.

## 6. Model predictive direct speed control with sliding mode load torque observer

**Fig 9:**
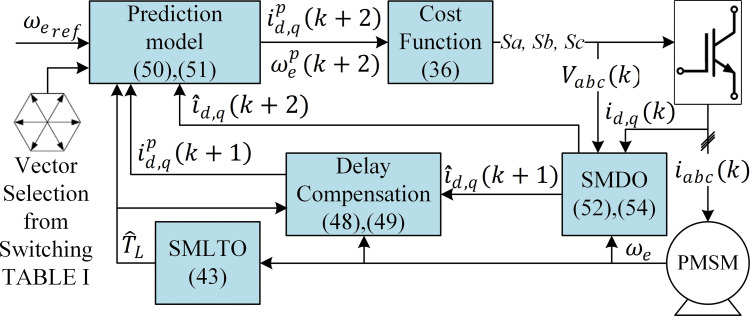
Block diagram of the model predictive direct speed control (MPDSC) approach.

MPDSC scheme is illustrated in Fig 9, where the predicted speed is directly incorporated into the cost function and compared to the reference speed. The corresponding optimization logic, shown in Fig 13, evaluates the following cost function at each sampling instant:

**Fig 10:**
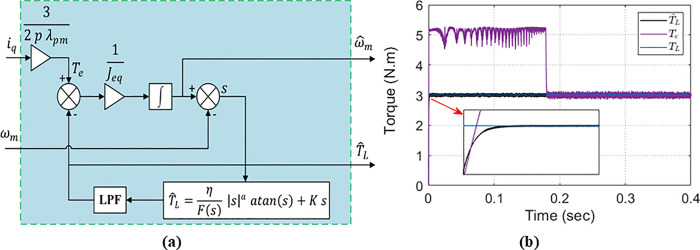
Sliding-mode load-torque observer (SMLTO): (a) schematic structure; (b) estimated vs. actual load torque during transient operation.

**Fig 11:**
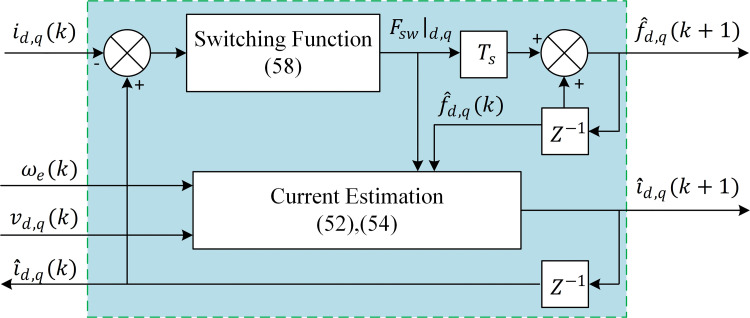
Sliding-mode disturbance observer (SMDO) for estimating stator current variation and compensating delay in MPDSC.

**Fig 12:**
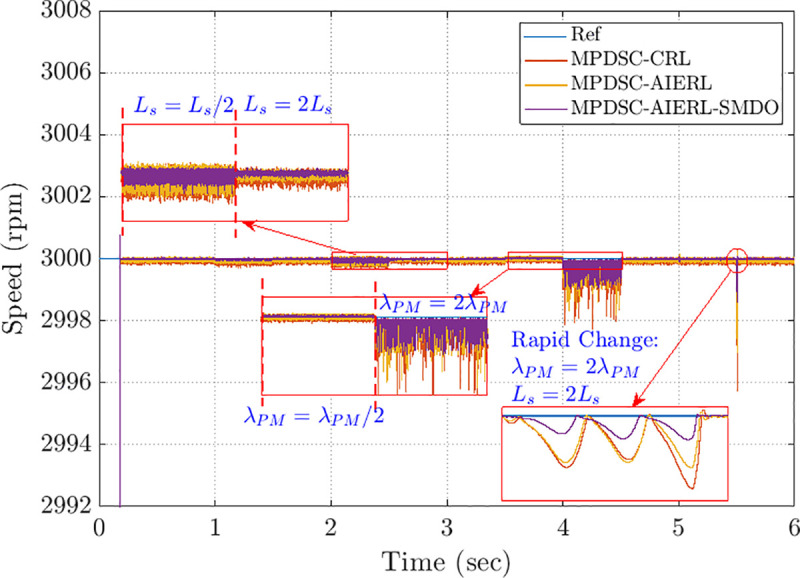
Comparison of controller performance during parameter-mismatch disturbances for MPDSC with and without adaptive reaching law/SMDO.


$$g=\;k_{i_{d}}\;{\left(\overset{p}{\underset{d}{i}}(k+\text{2})\right)}^{\text{2}}+k_{\omega }{\left(\overset{*}{\underset{e}{\omega }}-\overset{p}{\underset{e}{\omega }}(k+\text{2})\right)}^{\text{2}}+k_{i_{q}}{\left(\overset{*}{\underset{q}{i}}-\overset{p}{\underset{q}{i}}(k+\text{2})\right)}^{\text{2}}+f\left(\overset{p}{\underset{d}{i}},\overset{p}{\underset{q}{i}}\right)$$ (36)

where $\overset{p}{\underset{d,q}{i}}(k+\text{2})$are the predicted dq axes currents at instant (k+2); similarly $\overset{p}{\underset{e}{\omega }}(k+\text{2})\;$ is the speed prediction at instant (k+2); $\overset{*}{\underset{e}{\omega }}$ is the reference speed and $\overset{*}{\underset{q}{i}}$ is the reference quadrature-axis current obtained from (6) as follows:


$$\overset{*}{\underset{q}{i}}=\frac{\text{2}T_{e}}{\text{3}p\lambda_{PM}}.$$ (37)

In MPDSC, the electromagnetic torque $T_{e}$ is assumed equal to the estimated load torque ${\hat{T}}_{L}$ [51]; thus:


$$\overset{*}{\underset{q}{i}}=\frac{\text{2}{\hat{T}}_{L}}{\text{3}p\lambda_{PM}}.$$ (38)

The coefficients $k_{i_{d}},\;{\;k}_{i_{q}}$ and $k_{\omega }$ are weighting parameters. The last term is a nonlinear function amplitude-limiting function for the stator current, which can be expressed as:


$$\begin{array}{c} f\left(\overset{p}{\underset{d}{i}},\overset{p}{\underset{q}{i}}\right)=\;\left\{ \;\;\;\;\begin{array}{c} \;\text{0}\;\left|\overset{p}{\underset{d}{i}}\right|\leq i_{d\;max}\;and\;\left|\overset{p}{\underset{q}{i}}\right|\leq i_{q\;max}\\ \infty \;\left|\overset{p}{\underset{d}{i}}\right|>i_{d\;max\;}\;or\;\left|\overset{p}{\underset{q}{i}}\right|>i_{q\;max} \end{array}\right.\; \end{array}$$ (39)

To predict the mechanical angular speed [41] as stated in (10), the electromagnetic torque at the next sample time $\overset{p}{\underset{e}{T}}\;(k+\text{1})$ must be predicted. The predicted load torque ${\hat{T}}_{L}\;(k+\text{1})$ Is also required. Therefore, SMLTO shown in Fig 10(a) is used to estimate the load torque.

### 6.1 SMLTO for load torque observation

The sliding surface $s_{o}$ defined based on the speed estimation error as follows:


$$\begin{array}{c} s_{o}={\hat{\omega }}_{m}-\omega_{m} \end{array}$$ (40)

where ${\hat{\omega }}_{m}$ is the estimated mechanical speed and $\omega_{m}$ is the actual mechanical speed. Taking the Load Torque $T_{L}$ as the observation target, and treating $B_{m}$ as part of the load torque to be observed, the SMLTO dynamics are given by (41):


$${\dot{\hat{\omega }}}_{m}=\frac{\text{1}}{J_{eq}}(\frac{\text{3}}{\text{2}}p\;\lambda_{PM}i_{q}-{\hat{T}}_{L})$$ (41)

Substituting (41) and (7) in the derivative of (40), we can obtain (42):


$$\begin{array}{c} \dot{s_{o}}=\frac{\text{1}}{J}\left(-{\hat{T}}_{L}+T_{L}\right). \end{array}$$ (42)

When the system reaches equilibrium, $s_{o}=\text{0}$ implies $\dot{s_{o}}=\text{0}$; consequently, the real-time load torque, $T_{L}$, is equal to ${\hat{T}}_{L}$ as shown in (43).


$$\begin{array}{c} T_{L}={\hat{T}}_{L}=F_{sw}=\frac{\eta }{F\left(s_{o}\right)}\;{\left|s_{o}\right|}^{a}\;arctan\left(s_{o}\right)+K\;s_{o} \end{array}$$ (43)

In (43) the term $F_{sw}$ denotes the AIERL from (24) and (25). The arctan function is utilized in (43) to replace the sign function [52]. Fig 10(b) illustrates the SMLTO’s effectiveness: the estimated load torque closely tracks the load torque under disturbances and sudden step changes in load or speed

### 6.2. Delay compensation

FCS-MPC computational delays for PMSMs pose a significant challenge for accurate future-state prediction and maintaining stable control. The baseline FCS-MPC inherently includes one-step delay compensation through its single-step prediction mechanism. In contrast, the proposed MPDSC extends this concept by introducing a two-step compensation using the sliding-mode disturbance observer (SMDO) to further minimize current-prediction error at high speeds [53].

1. At first step, the variables$\;{\hat{i}}_{dq}$ at (k+1) are estimated using SMDO structure as in (52) and (54) (Section 6.4).
2. In the second step, the future values of $\overset{p}{\underset{dq}{i}}(k+\text{1})$ are calculated using the measured values at time step k and the estimated variables ${\hat{i}}_{d,q}(k+\text{1}),\;$ as shown in (48) and (49).
3. In the third step, ${\hat{i}}_{dq}(k+\text{2})$ are estimated in the same manner as ${\hat{i}}_{d,q}(k+\text{1})$. These estimates are then used to predict the stator current at time step k+2 as follows:

$$\overset{p}{\underset{d}{i}}(k+\text{1})=i_{d}(k)+\frac{T_{s}}{\text{2}L_{s}}\left[\text{2}v_{d}(k)-R_{s}\left(i_{d}(k)+{\hat{i}}_{d}(k+\text{1})\right)+\omega (k)L_{s}\left(i_{q}(k)+{\hat{i}}_{q}(k+\text{1})\right)\right]$$ (48)

$$\overset{p}{\underset{q}{i}}(k+\text{1})=i_{q}(k)+\frac{T_{s}}{\text{2}L_{s}}\left[\text{2}v_{q}(k)-R_{s}\left(i_{q}(k)+{\hat{i}}_{q}(k+\text{1})\right)-\omega (k)L_{s}\left(i_{d}(k)+{\hat{i}}_{d}(k+\text{1})\right)-\text{2}\omega (k)\lambda_{PM}\right]\;$$ (49)

$$\overset{p}{\underset{d}{i}}(k+\text{2})=\overset{p}{\underset{d}{i}}(k+\text{1})+\frac{T_{s}}{\text{2}L_{s}}\;\left[\text{2}v_{d_{i}}(k+\text{1})-R_{s}\left((\overset{p}{\underset{d}{i}}(k+\text{1})+{\hat{i}}_{d}(k+\text{2})\right)+\omega (k)L_{s}(\overset{p}{\underset{q}{i}}(k+\text{1})+{\hat{i}}_{q}(k+\text{2}))\right]$$ (50)

$$\overset{p}{\underset{q}{i}}(k+\text{2})=\overset{p}{\underset{q}{i}}(k+\text{1})+\frac{T_{s}}{\text{2}L_{s}}\;\left[\text{2}v_{q_{i}}(k+\text{1})-R_{s}\left((\overset{p}{\underset{q}{i}}(k+\text{1})+{\hat{i}}_{q}(k+\text{2})\right)-\omega (k)L_{s}\left(\overset{p}{\underset{d}{i}}(k+\text{1})+{\hat{i}}_{d}(k+\text{2})\right)-\text{2}\omega (k)\lambda_{PM}\right]$$ (51)

where $\overset{p}{\underset{e}{\omega }}(k+\text{1})$ is the predicted speed at the instant k+1 as in (10), and $v_{dq_{i}}$ denotes the basic inverter voltage vector of the synchronous rotating frame, selected to minimize the cost function stated in (36).

### 6.3. SMDO for Current Estimation

A sliding-mode disturbances observer based on the method in [54] is designed to estimate the disturbances arising from parameter mismatches online. Treating disturbances as extended states and starting from (8) and (9), the current dynamics are rewritten to include disturbance terms $f_{dq}(k)$, and their variation rates $F_{dq}(k)$.

The discrete-time SMDO for dq axes currents are formulated as follows:


$${\hat{i}}_{d}(k+\text{1})={\hat{i}}_{d}(k)+\frac{T_{s}}{L_{s}}\left(v_{d}(k)-R{\hat{i}}_{d}(k)+\omega (k)L_{s}i_{q}(k)-{\hat{f}}_{d}(k)+L_{s}{\;F_{sw}|}_{d}(k)\right)$$ (52)

$${\hat{f}}_{d}(k)={\hat{f}}_{d}(k+\text{1})-T_{s}\;g_{d}\;{\;F_{sw}|}_{d}(k)$$ (53)

$${\hat{i}}_{q}(k+\text{1})={\hat{i}}_{q}(k)+\frac{T_{s}}{L_{s}}({v_{q}(k)-R\hat{i}}_{q}(k)\;\;+\omega_{e}(k)\left(L_{s}{\hat{i}}_{d}(k)+\lambda_{pm}\right)-{\hat{f}}_{q}(k)+{\;F_{sw}|}_{q}(k))$$ (54)

$${\hat{f}}_{q}(k)={\hat{f}}_{q}(k+\text{1})-T_{s}\;g_{q}\;{\;F_{sw}|}_{q}(k)$$ (55)

where:

- $f_{dq}(k)$ is defined as an unknown disturbance cause the current variation,
- ${\hat{f}}_{dq}(k)$ are its estimate,
- $F_{dq}(k)$ is the variation rate of these disturbances,
- ${\hat{i}}_{dq}(k+\text{1})$ are the estimated variables of stator current dq components in the instant (k+1),
- $v_{dq}(k)\;$ are the applied voltage vectors at time k,
- ${\;F_{sw}|}_{dq}$ are components of the sliding mode switching function, for simplicity Constant Reaching Law (CRL) defined in (58) is employed,
- $g_{dq}$ are a coefficient greater than zero.

The sliding surface is defined as the current estimation error:


$$s_{smdo}=e_{dq}(k)={\hat{i}}_{dq}(k)-i_{dq}(k).\;$$ (56)

Subtracting the original model in (8) and (9) from observer model in (52) and (54) with neglecting the error of disturbance estimation $\left({\hat{f}}_{dq}-f_{dq}=\text{0}\right)$, gives:


$${\dot{e}}_{dq}=-\frac{R}{L}e_{dq}+\omega (k)e_{qd}-\frac{F_{sw}{\;|}_{dq}}{L}$$ (57)

Applying the Constant RL and solving for $F_{sw}{\;|}_{dq}$ yields:


$$F_{sw}{\;|}_{dq}={-k}_{dq}\;sign(s_{smdo})$$ (58)

The SMDO block diagram is illustrated in Fig 11. The proposed SMDO enables accurate online estimation of stator currents and disturbance forces, improving MPC predictive accuracy and effectively compensating for inherent system delays

To assess the effect of adding the SMDO to MPDSC; a parameter-sensitivity study was performed for three approaches:

1. MPDSC without SMDO, using traditional delay compensation and a constant RL as proposed in [55].
2. MPDSC without SMDO, with AIERL employed in SMLTO.
3. MPDSC with SMDO, and AIERL employed in SMLTO.

Fig 12 compares the three control approaches of the steady state error under several abrupt parameter changes.

At different time intervals, the motor parameters are deliberately varied to evaluate robustness:

- 2–3 s: Stator inductance $L_{s}$is halved ($L_{s}=L_{s}/\text{2}$) and doubled ($L_{s}=\text{2}L_{s}$) to test inductance sensitivity.
- 3.5–4.5 s: Permanent magnet flux linkage $\lambda_{PM}$ is reduced to half and then doubled ($\lambda_{PM}=\lambda_{PM}/\text{2}$, $\lambda_{PM}=\text{2}\lambda_{PM}$) to simulate magnetic flux weakening and strengthening.
- After 5.5 s: A rapid change occurs where both $\lambda_{PM}=\text{2}\lambda_{PM}$ and $L_{s}=\text{2}L_{s}$, testing the transient adaptation capability of each controller.

The zoomed insets highlight the steady-state and transient speed behaviors under each disturbance. The MPDSC-AIERL-SMDO method exhibits the smoothest and most stable speed tracking with minimal oscillation and rapid recovery, confirming its superior robustness and adaptability compared to MPDSC-AIERL and MPDSC-CRL.

Table 3 represents numerical results (Mean, CV and SD of speed ITAE) for 100 randomized parameter draws with ±50% variations in $R_{s}$, $L_{s}$ and $\lambda_{PM}.$

**Table 3:** Comparison of parameter-mismatch impact on MPDSC variants, showing mean, standard deviation (SD), and coefficient of variation (CV) of ITAE across 100 randomized trials.

	MPDSC – CRL (No SMDO)	MPDSC – AIERL (No SMDO)	MPDSC – AIERL (with SMDO)
Mean ITAE	37.40	10.61	11.77
CV	2.06	1.53	1.38
SD	76.9168	16.2008	16.2796

### 6.4. Stability analysis

Theorem 2: Stability of the proposed MPDSC with sliding-mode observers

Consider the PMSM system controlled by the proposed MPDSC incorporating the SMLTO defined in (41)-(43) and SMDO defined in (52)-(55).

Assume that:

1. The SMLTO reaching law employs the AIERL with parameter satisfies K,η,b>0 and 0<a,m<1 and F(s)≥0.
2. The SMDO sliding gains are strictly positive and bounded.
3. The PMSM parameters and disturbances are bounded and the stator currents and speed remain bounded under closed-loop operation.

Then, the load-torque estimation error and current-prediction error converge asymptotically to zero, and the overall MPDSC closed-loop system is stable with bounded speed-tracking error.

Proof

Consider the Lyapunov candidate function


$$V_{o}=\frac{\text{1}}{\text{2}}\overset{\text{2}}{\underset{o}{s}}$$ (44)

with $V_{o}(\text{0})=\text{0}$ and $V_{o}(t)>\text{0}$ for $s_{o}(t)\neq \text{0}$. Then the reaching condition for the observer is:


$$\dot{V_{o}}=s_{o}\;\dot{s_{o}}<\text{0}$$ (45)

Substituting (42) into (45) yields (46)


$$\dot{V_{o}}=s_{o}\left(\;\frac{\text{1}}{J}\left(-{\hat{T}}_{L}+T_{L}\right)\right)=s_{o}\left(\;\frac{\text{1}}{J}\left(-\frac{\eta }{F\left(s_{o}\right)}\;{\left|s_{o}\right|}^{a}\;sgn\left(s_{o}\right)-K\;s_{o}\right)\right).\;$$ (46)

So, the Lyapunov derivative becomes as shown in (47):


$$\dot{V_{o}}=-\frac{\eta }{J\;F\left(s_{o}\right)}{\left|s_{o}\right|}^{a+\text{1}}-\frac{K}{J}\;\overset{\text{2}}{\underset{o}{s}}$$ (47)

Considering the constraints for parameter tuning for K, η, b>0 and 0<a,m<1, for all $s_{o}\neq$0, $\dot{V_{o}}$< 0 which the load-torque estimation error converges asymptotically to zero and accurate torque estimation.

For the SMDO, the current-estimation error sliding surface defined in (56) satisfies a standard sliding-mode reaching condition under strictly positive gains, guaranteeing asymptotic convergence of the current-prediction error.

Since the MPDSC cost function in (36) relies on bounded predicted speed and current estimates, and both SMLTO and SMDO errors converge asymptotically, the closed-loop MPDSC system is stable with bounded speed-tracking error.

## 7. Weighting factors and control parameters tuning

In [56], methods for selecting cost function weighting factors in Model Predictive Control (MPC) strategies are classified into:

- Traditional approaches, which utilize trial-and-error tuning,
- Numerical and algebraic methods that adjust weighting factors to system conditions (for example variations in speed or current), and
- Alternative methodologies that employ optimization and artificial intelligence for real-time adaptive tuning.

This work addresses the cost-function weighting factors as related to the controller parameters, and formulates their selection as an offline multi‑objective optimization problem. The employed optimization strategy combines Gradient Descent (GD) with the Sequential Quadratic Programming (SQP) algorithm, implemented using the Response Optimizer tool in Simulink/MATLAB—a commonly used tool for tweaking control system settings to attain optimal system performance.

- The GD Method is a first-order optimizer that iteratively modifies control parameters in the direction of the negative objective function gradient to reduce the discrepancy between desired and actual responses.
- The SQP algorithm is a second-order optimization method that employs a quadratic approximation of the objective function to find ascertain optimal parameter values.

These methods assist in identifying parameter values that ensure the control system attains the intended performance. The Response Optimizer also supports other techniques such as evolutionary algorithms and pattern search.

## 8. Simulation and results

### 8.1. Description of the simulation environment

The three control schemes—PI-MPCC, SMSC-MPCC, and MPDSC—were verified through simulation research conducted in MATLAB/Simulink environment. Motor parameters are presented in Table 4.

**Table 4:** Motor specifications.

Motor Parameter	Symbol	Values	Units
Stator Resistance	$R_{s}$	2.875	Ω
Stator Inductance	$L_{s}$	8.5	mH
Magnet flux linkage	$\lambda_{pm}$	0.175	V/rad/s
Moment of inertia	J	0.8*10−3	$kg.m^{\text{2}}$
Total Moment of inertia	$J_{eq}$	2.4*10−3	$kg.m^{\text{2}}\;$
Viscous damping	$B_{m}$	0.001	N·m.s
Pair of Poles	p	2	
Rated Speed		3000	RPM
Rated Torque		3	N·m
DC Voltage		220	V
Rated Current/ Max. Current		4/6	A
Rated Power		1.1	kW
Frequency		50	Hz

Each control method was verified under two operational conditions: loading and unloading, as well as under speed step-up and step-down.

The motor reference speed is shown in Fig 14a

- Commenced at 1500 rpm for 1 second,
- increased to the rated speed of 3000 rpm from 1 second to 2.5 seconds,
- decreased to 1000 rpm from 3 seconds to 4 seconds.
- At 4 seconds, the motor ceased operation at a reference speed of zero, remaining inactive until the conclusion of the simulation at 5 seconds.

The applied load torque, illustrated in Fig 14(b):

- Commenced at 1.5 N·m and
- transitioned to the rated load torque of 3N·m between 0.5 s and 3.5 s.
- At 3.5 s, the load was reduced to 1 N.m, leading to no-load operation by 4.5 s, which continued until 5 s,
- then low speed applied at 150 rpm at the rated torque adjusted again to 3 N·m.

The Model Predictive Controller (MPC) sampling time is 20 μs, applied to a two-level full bridge voltage source inverter with DC input voltage of 220 V.

### 8.2 Speed Tracking Evaluation

The reference speed profile includes multiple levels that require swift tracking and stabilization for each control method. Furthermore, variations in torque load at different levels necessitate insensitivity to load disturbances. Fig 15 illustrates the speed-tracking results for the three control approaches compared to the reference speed.

Five detailed close views of operational transient responses, labelled (1), (2), (3), (4) and (5), are presented in Fig 16(a), (b), (c), (d) and (e) to facilitate detailed comparison of transient behavior.

**Fig 13:**
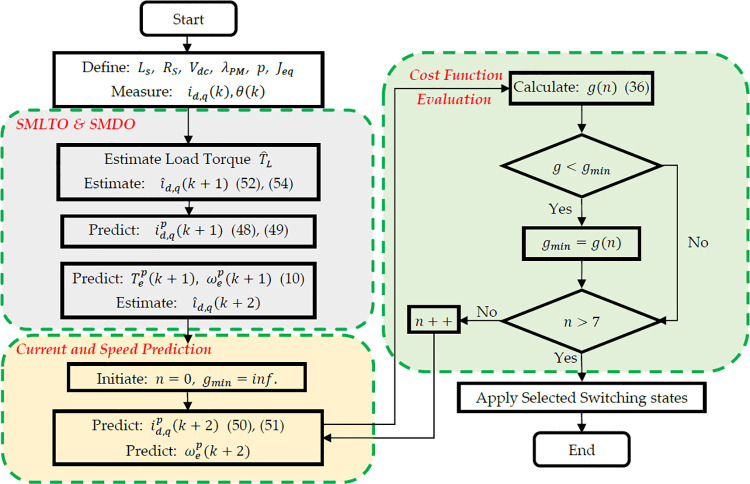
Flowchart of the MPDSC process integrating SMLTO and SMDO for current and speed prediction.

**Fig 14:**
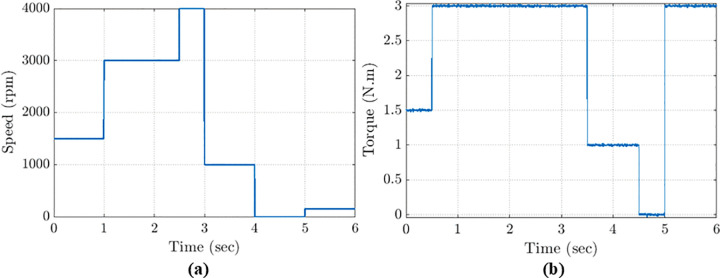
Reference speed and mechanical load-torque profiles used in all simulation scenarios: (a) speed trajectory; (b) applied torque.

**Fig 15:**
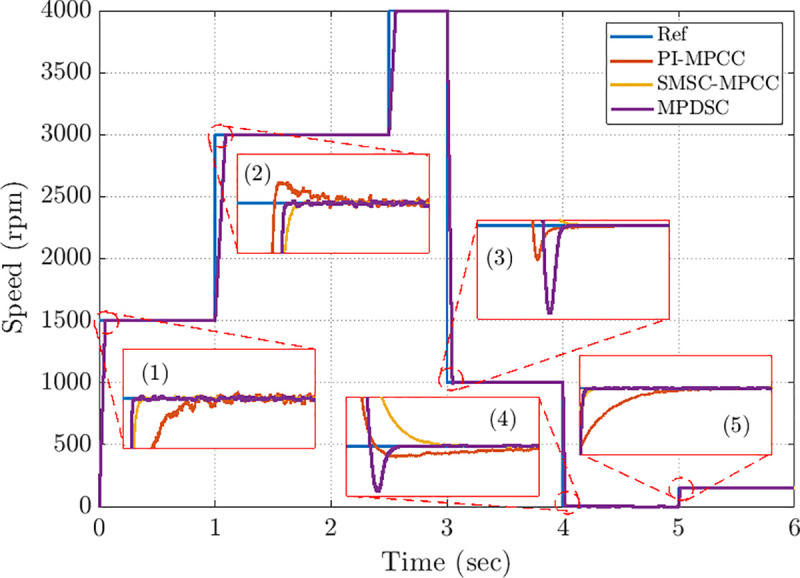
Speed-tracking responses under PI-MPCC, SMSC-MPCC, and MPDSC control across various operating points.

**Fig 16:**
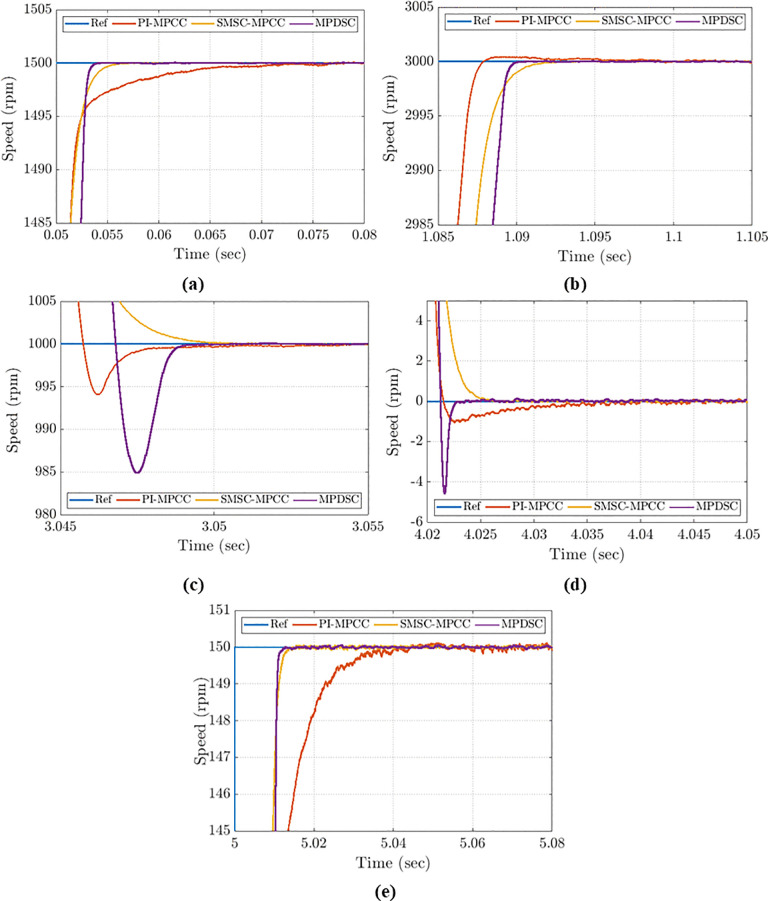
Detailed speed-evaluation comparison for all control approaches: (a) startup; (b) speed step-up; (c) speed step-down; (d) stopping under no-load; (e) low-speed operation.

#### 8.2.1. The performance of PMSM in startup conditions.

Fig 16(a) presents the startup transient speed for the three control approaches with an initial reference speed of 1500 rpm and a load torque of 1.5 N·m. MPDSC demonstrates the fastest response without any overshoot. The settling times recorded for PI-MPCC, SMSC-MPCC, and MPDSC are: 0.073 s, 0.056 s, and 0.053 s, respectively.

#### 8.2.2. Speed Step up transient performance.

Fig 16(b) compares the three control approaches when the reference speed is increased to the rated speed of 3000 rpm. MPDSC and SMSC-MPCC both demonstrate rapid stabilization without overshoot and exhibit minimal steady-state error, reaching the reference speed in 90 ms and 93 ms, respectively. The average steady-state values for PI-MPCC, SMSC-MPCC and MPDSC are: 0.4 rpm, 0.2 rpm, and 0.17 rpm, respectively.

#### 8.2.3. Transient performance during speed step down.

Fig 16(c) presents the transient speed performance when the setpoint is reduced from 4000 rpm to 1000 rpm. SMSC‑MPCC performs effectively with no undershoot and a settling time of 51 ms. PI‑MPCC exhibits an undershoot of ≈6 rpm and a settling time of 55 ms. MPDSC is the most aggressive: it shows a 15 rpm undershoot but the shortest settling time of 49 ms.

#### 8.2.4. Stopping performance during no-load operation.

Fig 16(d) illustrates the transient speed performance when the reference speed is reduced from 1000 rpm to 0 rpm under no‑load conditions.

- PI-MPCC reaches the zero speed at the longest settling time (40 ms) and with a notable undershoot (1 rpm).
- MPDSC reaches the zero speed at the largest undershoot (4.5 rpm) and with the shortest settling time (22.5 ms).
- SMSC-MPCC gives the most balanced behavior: no undershoots and a settling time of (26 ms).

#### 8.2.5. Low-speed operation at 150 rpm (5% of the rated speed).

Fig 16(e) shows the response when the reference is stepped to 150 rpm (5% of the rated speed) at the rated torque. MPDSC and SMSC-MPCC have similar settling time at 10 ms, while PI-MPCC is slower (45 ms). Peak-to-peak speed oscillations for PI-MPCC, SMSC-MPCC and MPDSC are: 0.47 rpm, 0.2 rpm and 0.16 rpm, respectively.

### 8.3. Assessment of disturbance rejection

#### 8.3.1. Disturbance rejection ability when the applied load torque is increased.

**Fig 17:**
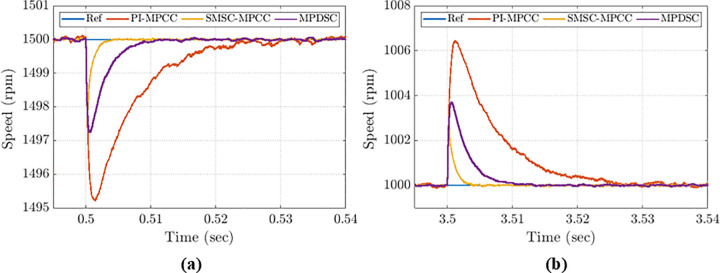
Disturbance rejection under load-torque variation: (a) torque increase; (b) torque decrease.

Fig 17(a) illustrates the disturbance rejection capabilities of each control method when the load torque jumps from 1.5 Nm to 3 Nm. Both MPDSC and SMSC-MPCC recover to the reference speed in roughly 10 ms. Conversely, PI-MPCC exhibits the largest undershoot of 5 rpm and the longest settling time of 30 ms.

#### 8.3.2. Disturbance rejection ability when decreasing the applied load torque.

Fig 17(b) shows the response when the load torque falls from 3 Nm to 1 Nm. SMSC-MPCC has the fastest recovery time at 4 ms, while MPDSC and PI-MPCC have recovery time of 10 ms and 30 ms respectively. Moreover, SMSC-MPCC has the lowest overshoot of 0.25% of the reference speed while MPDSC and PI-MPCC examined 0.38% and 0.64%, respectively. These results indicate that SMSC-MPCC has the most superior performance in terms of disturbance rejection.

### 8.4. Assessment of torque stability

**Fig 18:**
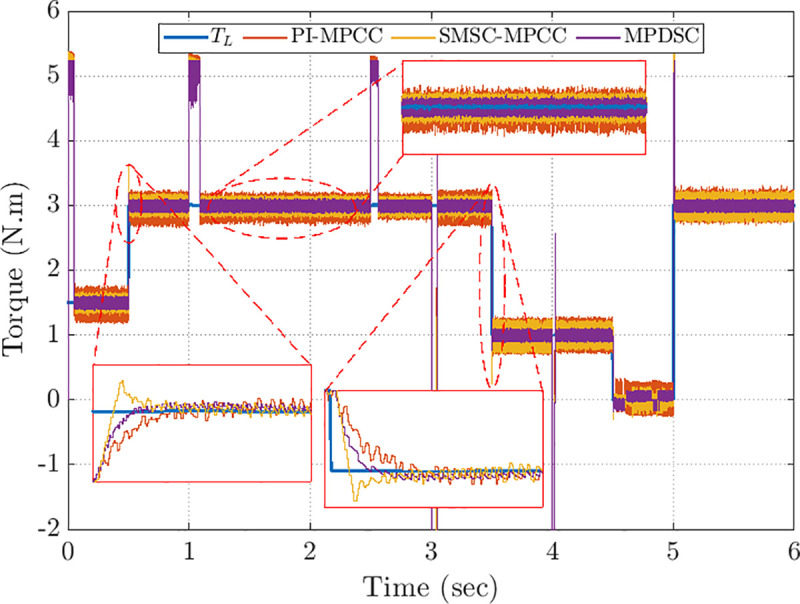
Torque responses of the three controllers during load changes.

Fig 18 depicts the developed torque, $T_{e}$, during sudden load torque variations for PI-MPCC, SMSC-MPCC, and MPDSC control approaches. MPDSC exhibits the highest efficiency in response, characterized by almost no overshoots and the fastest stabilization during machine loading and unloading. Under steady-state conditions, peak-to-peak oscillations for PI-MPCC, SMSC-MPCC, and MPDSC are around: 0.67 Nm, 0.41 N·m, and 0.28 N·m, respectively. Consequently, MPDSC exhibits the most consistent and stable performance with the smallest ripples.

### 8.5 Assessment of Harmonics

**Fig 19:**
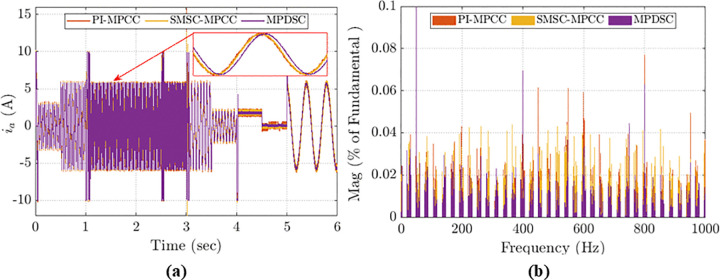
(a) Stator current in phase-a under sudden speed and load changes; (b) total harmonic distortion (THD %) comparison among control schemes.

Figure 19(a) shows the phase-a stator current response to sudden changes in load and speed for the three control approaches. Under rated parameters for speed and torque operation, the total harmonic distortion (THD%) for PI-MPCC, SMSC-MPCC, and MPDSC are: 4.67%, 4.8%, and 2.27%, respectively, as shown in Fig 19(b). In [56], the authors simulated the SMSC, mentioned in [18], using Super Twisting Algorithm (STA) which is a RL that combines an integral term of the sliding surface with PRRL, to reduce chattering effect. Their results show that using that STA reduced THD% by almost 35% compared with the conventional SMSC-MPCC. In this paper, the results shows that MPDSC reduces THD% by almost 47%, indicating improved drive efficiency.

### 8.6. Evaluation of Idq currents

**Fig 20:**
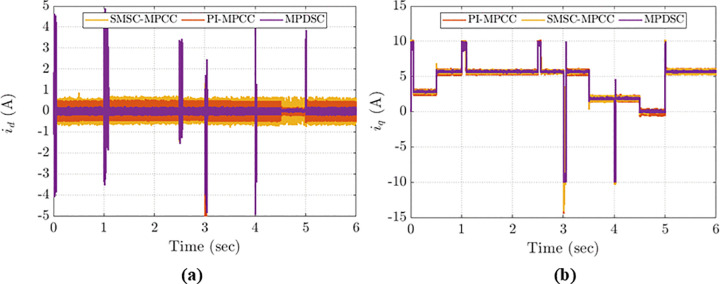
d- and q-axis current responses under dynamic operating conditions: (a) $i_{d}$ currents; (b) $i_{q}$ currents.

Fig 20 depicts the transient and steady-state performance of the $I_{dq}$currents during sudden load and speed variations. Key observations:

- Disturbances in $I_{dq}$currents reflect the motor’s ability to maintain speed under load changes. Large disturbances typically cause slower speed responses and can lead to instability.
  - When $I_{dq}$ get off track, the motor consumes more energy and heats up, which is not efficient for long-term use.
  - MPDSC attains minimal oscillations in steady state operation

### 8.7. $V_{dq}$ Assessment

**Fig 21:**
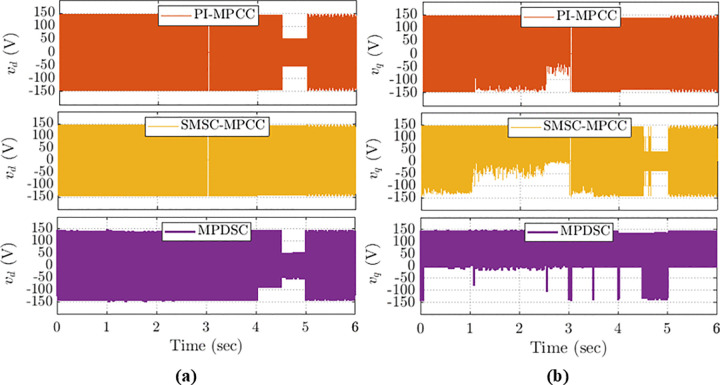
Voltage response under abrupt load variations: (a) ${𝐯}_{d}$ component; (b) ${𝐯}_{q}$ component for all control schemes.

Fig 21 shows the stator quadrature-axis and direct-axis voltages across several simulated situations for the three control strategies: PI-MPCC, SMSC-MPCC, and MPDSC.

- MPDSC consistently shows a favorable behavior: voltages are smoother and exhibit smaller oscillations, which correlates with a more stable and less oscillatory torque response (as seen in the torque analysis).
- PI-MPCC displays pronounced oscillations, which correspond to larger torque ripples.
- A predominance of positive, smoother voltage waveforms indicates reduced switching activity and therefore lower inverter switching losses.
  - All three controllers show broadly similar analogue responses, but MPDSC has markedly lower amplitude during no‑load operation, indicating improved stability and reduced losses in that condition.

The comparative analysis of PI-MPCC, SMSC-MPCC, and MPDSC control methodologies applied to the PMSM demonstrates that:

- MPDSC regularly surpasses the other techniques in:
  - speed tracking, disturbance rejection, torque response, and overall system stability.
  - diminished torque ripples, and the lowest total harmonic distortion percentage.
  - enhancing efficiency and minimizing inverter losses.
- SMSC-MPCC demonstrates:
  - Good steady-state accuracy
  - Slightly weaker dynamic performance compared with MPDSC.
- PI-MPCC frequently exhibits:
  - elevated overshoot, longer settling times, and increased oscillations in both speed and torque regulation,
  - rendering PI-MPCC to be the least advantageous of the three options.

The findings demonstrate that MPDSC is the most efficient control strategy for PMSM applications necessitating rapid reaction and minimal energy losses.

### 8.8. Performance evaluation under parameter mismatch

To assess robustness, many tests of machine parameter mismatches were performed for the three control approaches. The study focused on how variations in PMSM parameters—particularly stator inductance and permanent-magnet flux linkage—affect prediction accuracy and performance. All tests began from the same initial conditions and were run under rated operating conditions. Parameter alterations were applied every 0.5s. Performance data statistics of the machine under rated operating conditions are shown in Table 5.

**Table 5:** Motor statistics at rated operating conditions comparing torque, current, and THD % among controllers.

		MPDSC	SMSC-MPCC	PI-MPCC
**THD%**		2.28	4.80	4.67
$T_{e}\;$(N·m)	Average	3	3	3
	Peak to peak	0.28	0.41	0.67
$i_{d}\;$(A)	Average	0	0.03	0.01
	Peak to peak	0.46	1.44	1.03
$i_{q}\;$(A)	Average	5.717	5.717	5.717
	Peak to peak	0.47	0.72	0.96

**Fig 22:**
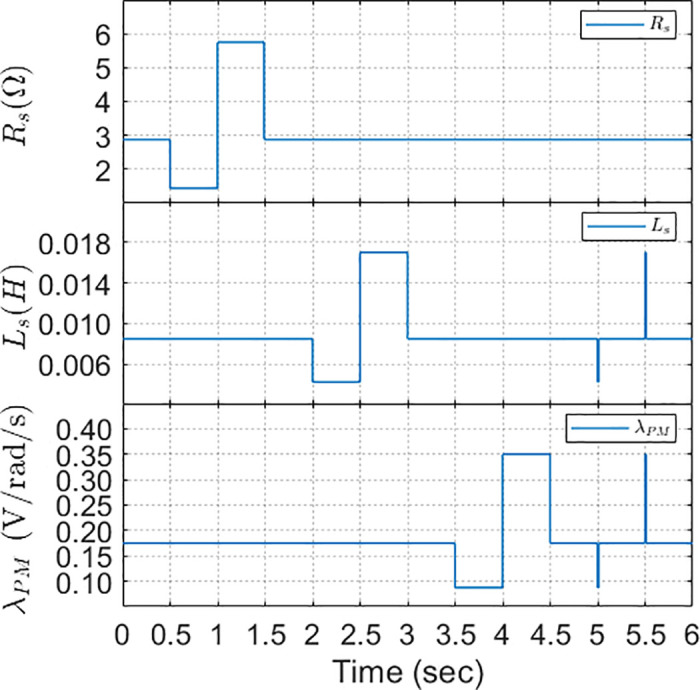
Online variation of machine parameters (${𝐑}_{s},L_{s},\lambda_{PM}$) during operation at rated conditions.

The simulated scenarios involved changing stator resistance from the initial value ${\overset{´}{R}}_{s}$ to 0.5${\overset{´}{R}}_{s}$ and 1.5${\overset{´}{R}}_{s}\;$at various intervals. Analogue measurements were conducted for stator inductance and equivalent variations for flux linkage. Simultaneous reduction and growth of $L_{s}$ and $\lambda_{PM}$ are shown in Fig 22.

#### 8.8.1. Simulation of stator resistance variation.

**Fig 23:**
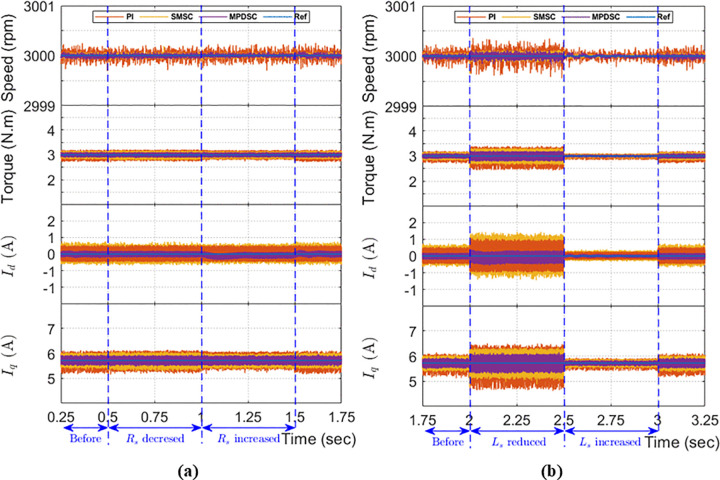
Comparative performance under parameter mismatch: (a) stator resistance variation; (b) stator inductance variation.

Fig 23(a) illustrates the response to step changes in resistance at 0.5 s (50% decrease) and at 1.0 s (50% increase). All three control strategies—PI-MPCC, SMSC-MPCC, and MPDSC—exhibit nearly identical behavior in speed, torque, and stator currents. This indicates that each controller effectively handles resistance variations with similar disturbance-rejection capabilities.

#### 8.8.2. Simulation of stator inductance variation.

Fig 23(b) shows the behavior when stator inductance is reduced by 50% of its initial value, ${\overset{´}{L}}_{s},$ at 2.0 s and then increased to $\text{1}.\text{5}{\overset{´}{L}}_{s}$ at 2.5 s. The system remains stable under all three control strategies, but MPDSC demonstrates enhanced performance with smaller ripples and less oscillations in speed, torque, and current. The total harmonic distortion (THD%) analysis further supports this conclusion as follows:

- When the inductance is reduced, the THD% values for SMSC-MPCC, PI-MPCC and MPDSC are 9.67%, 9.33% and 4.6%, respectively.
- Upon increasing the inductance, the corresponding THD% values improve (decrease) to 2.39%, 2.33%, and 1.19%, respectively. Consequently, MPDSC consistently attains the lowest THD%.

#### 8.8.3. Simulation under PM Flux-linkage mismatch.

**Fig 24:**
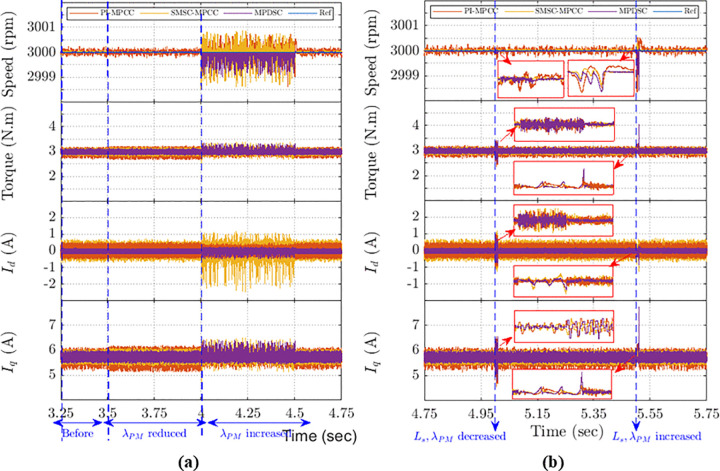
PMSM performance under flux-linkage and inductance mismatch: (a) flux-linkage variation; (b) simultaneous flux-linkage and inductance changes.

Fig 24a shows the speed, torque and currents responses when flux linkage is decreased to 50% of its initial value, ${\overset{´}{\lambda }}_{PM}$, at 3.5 s and then increased to 1.5${\overset{´}{\lambda }}_{PM}$ at 4 s. MPDSC produces the lowest ripples in speed, torque, and currents, although it exhibits aggressive spikes during each transient immediately after each sudden flux linkage variation. The transients arise because the load-torque observer uses flux linkage feedback; sudden flux changes temporarily perturb the estimate until the observer adapts. Despite these aggressive transients, MPDSC controls the amplitude of $I_{dq}$disturbances better than SMSC‑MPCC and PI‑MPCC and delivers superior steady‑state disturbance rejection. Fig 24a confirms that MPDSC outperforms SMSC-MPCC and PI-MPCC in parameter mismatch disturbance rejection in the steady state despite the aggressive transient response.

#### 8.8.4. Simulation under rapid change of stator inductance and flux linkage.

Fig 24(b) illustrates the system response when $L_{s}$ and $\lambda_{PM}$ are simultaneously pulsed to half of their initial values, i.e., $L_{s}=\text{0}.\text{5}{\overset{´}{L}}_{s}$ and $\lambda_{PM}=\text{0}.\text{5}{\overset{´}{\lambda }}_{PM}$. Then the system followed by restoration to their normal values before it experienced another rapid change of increase.

The results demonstrate that: MPDSC exhibits better steady state performance with lower speed, torque and currents ripples. However, MPDSC shows noticeable transient spikes which is attributed to the sensitivity of the load torque estimation based on sliding mode observation.

These aggressive transients evident in speed, torque and current responses since these rapid variations cause oscillations and longer settling time, which is about (15–20 ms). In contrast, SMSC-MPCC followed by PI-MPCC show smoother transient behavior but suffer from higher steady state ripples.

#### 8.8.5. Parameter sensitivity analysis.

**Fig 25:**
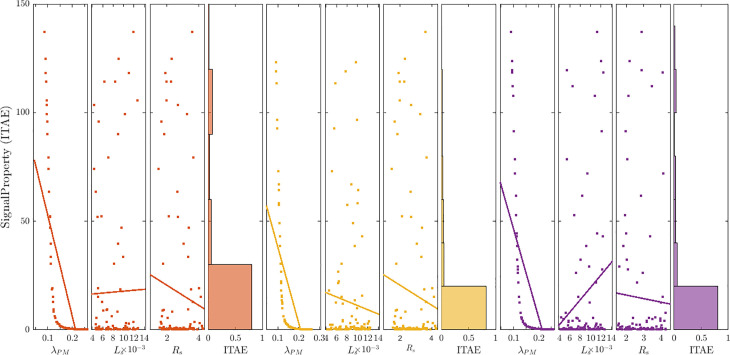
Sensitivity of ITAE to parameter mismatch for PI-MPCC, SMSC-MPCC, and MPDSC controllers.

To identify the most robust method against the motor parameter variations, a parameter sensitivity analysis was performed for the three control approaches. Fig 25 shows scatter plots that visualize how parameter variations affect the motor speed Integral Time Absolute Error (ITAE). Table 6 summarizes the dispersion statistics.

**Table 6:** ITAE dispersion under parameter mismatch (±50% Monte Carlo, N = 100) for PI-MPCC, SMSC-MPCC, and MPDSC controllers.

	PI-MPCC	SMSC-MPCC	MPDSC
Mean	17.40602	12.75624	14.65119
SD	35.09595	27.43796	32.00042
CV	2.016311	2.150945	2.184151
Minimum	0.177048	0.169965	0.187661
Maximum	137.2493	123.0864	137.2162

- Sensitivity coefficients were obtained from 100 Monte Carlo trials,
- each trial sampled ($\lambda_{PM}$, $L_{s}$, $R_{S}$) independently from uniform distributions spanning ±50% around their nominal values (${\overset{´}{\lambda }}_{PM}$, ${\overset{´}{L}}_{s}$, ${\overset{´}{R}}_{S}$),
- ITAE was computed per each trial and used for correlation and ranking analyses.

Key findings:

- Across all three controllers, $\lambda_{PM}$ dominates the sensitivity of ITAE: correlations are strongly negative: MPDSC ≈ −0.65, SMSC-MPCC ≈ −0.63, PI-MPCC ≈ −0.70. In other words, larger $\lambda_{PM}$ tends to reduce error.
- $L_{s}$has weak and approach-dependent influence, MPDSC shows a slight increase in ITAE with higher Ls, r ≈ +0.22, in SMSC-MPCC a slight decrease, r ≈ −0.08 and in almost no influence PI-MPCC ~0.
- Rs is only mildly negative, about (−0.04 to −0.10 across all methods), indicating minimal direct effect on ITAE within the tested range.
- SMSC-MPCC exhibit the lowest mean ITAE and Standard Deviation (SD), indicating better average tracking and the tightest absolute spread-hence the highest practical robustness.
- MPDSC follows SMSC‑MPCC, and PI‑MPCC ranks last in mean ITAE.
- PI-MPCC has the lowest coefficient of variation (CV), implying lower relative variability, but this is accompanied by a higher mean ITAE and a more pronounced dependence on $\lambda_{PM}$ variation. This is likely due to its reliance on accurate direct time model prediction without intermediate current loops.
  - Flux-linkage mismatch induces errors in quadrature current prediction, which propagates to torque estimation and causes selection of suboptimal finite-set voltage vectors thereby elevates speed error (ITAE).

Supporting literature and interpretation:

- Reference [57] reports that the current prediction error is assessed against the parameter mismatch impact. For d-axis current, it is mainly affected by stator inductance with little impact from stator resistance and non from magnetic flux. For q-axis current, the error is strongly affected by magnetic flux, moderately by stator inductance and slightly by stator resistance. These parameter mismatches significantly affect the current prediction.
- The previous results support these findings and show the essential need for an observer to detect errors as well as providing real-time compensation to improve robustness.

**Fig 26:**
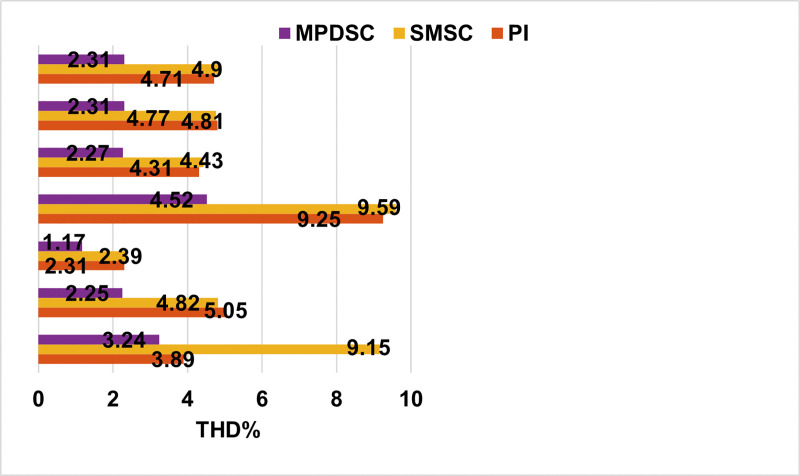
Total harmonic distortion (THD %) comparison of the three controllers under various parameter-mismatch scenarios.

However, SMSC-MPCC is more robust against the inductance and flux linkage mismatch disturbances with acceptable fluctuations. In contrast, MPDSC exhibits the lowest THD% levels overall the parameter mismatch scenarios compared to the other control methods as shown in Fig 26. These results highlight that, despite the transient sensitivity to flux linkage mismatch disturbances, MPDSC remains the most effective method achieving high performance control with minimal ripples and better steady-state accuracy.

### 8.9. Frequency domain response

**Fig 27:**
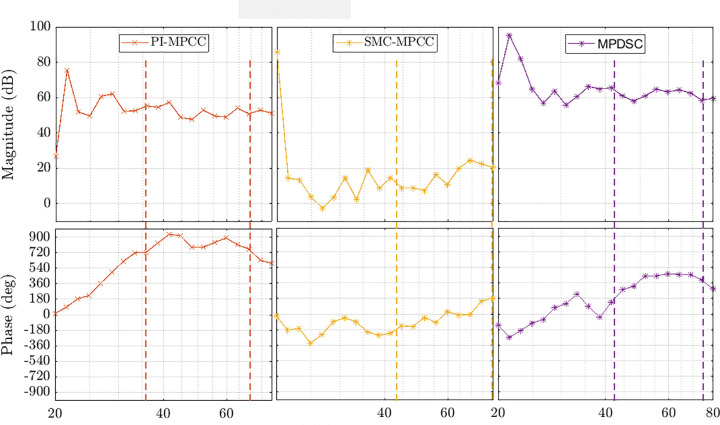
Bode diagrams comparing frequency domain stability margins of PI-MPCC, SMSC-MPCC, and MPDSC in the 20–80 Hz range.

From the Bode diagrams shown in Fig 27, the closed-loop stability windows are:

- PI-MPCC ≈ 36–70 Hz,
- SMSC-MPCC ≈ 43–80 Hz, and
- MPDSC ≈ 42–75 Hz.

Within these bands the loop has robust and non-oscillatory behavior. SMC exhibits the widest stable range, MPDSC is intermediate, and PI-MPCC is the narrowest. Outside these ranges the Bode plots show shrinking phase margin and gain peaking, indicating the onset of oscillation behavior or instability.

## 9. Discussion

The following discussion synthesizes the comparative results and highlights practical design insights derived from the conducted simulation studies. We evaluated two predictive sliding-mode PMSM speed controllers: SMSC-MPCC with a co-adaptive design (ASMS+AIERL) and MPDSC with SMLTO and SMDO for sensorless operation and delay compensation. Using a deterministic, scenario-controlled protocol (separate models, identical solver/sampling, scripted disturbances/parameter profiles, fixed noise), we benchmarked performance for step/load changes, low-speed operation (5% rated), parameter-mismatch, and frequency-sweep tests.

Summary of the findings:

- SMSC-MPCC delivered the lowest average tracking error, the widest stability band, and reduced chattering
- MPDSC achieved the fastest disturbance recovery while remaining sensorless and provided superior THD% when that metric is prioritized.
- SMSC-MPCC (ASMS+AIERL) is preferred when robustness and low chattering are paramount, whereas MPDSC (SMLTO+SMDO) is favored for sensorless operation, rapid disturbance rejection, and low THD%.

These results are simulation-based and assuming a standard drive model: ideal inverter, no core/eddy losses, and nominally constant parameters except in mismatch studies.

Future work will include:

- Bench hardware validation,
- Embedded (DSP/MCU/FPGA) profiling with fixed-point effects,
- Incorporation of inverter and magnetic non-idealities (dead-time, device drops/saturation, quantization/latency, thermal/magnetic drift), and
- Exploration of learner-in-the-loop auto-tuning for ASMS/AIERL or SMDO within real-time budgets.

In addition, recent leakage-type adaptive observer concepts could complement the sliding-mode disturbance observer (SMDO) structure adopted in this work [58], offering a potential hybrid design direction for future studies.

## Supporting information

S1 File Data Availability.zip, ZIP archive with 6 Datasets of Monte Carlo simulation results, speed ITAE sensitivity data (.xlsx), 27 figures (600 dpi TIFF), 6 CSV tables, MATLAB/Simulink models and Text file with usage instructions, file descriptions, and contact info. (ZIP)

## Abbreviations

PMSM: Permanent Magnet Synchronous Motor
PI-MPCC: Proportional-Integral based Model Predictive Current Controller
SMSC-MPCC: Sliding Mode Speed Control based Model Predictive Current Control
MPDSC: Model Predictive Direct Speed Control
SMC: Sliding Mode Control
SMLTO: Sliding Mode Load Torque Observer
SMDO: Sliding Mode Disturbance Observer
ASMS: Adaptive Sliding Mode Surface
CSMS: Conventional Sliding Mode Surface
FOC: Field Oriented Control
DTC: Direct Torque control
PWM: Pulse Width Modulation
THD: Total Harmonic Distortion
MPC: Model Predictive Control
FCS-MPC: Finite Control Set Model Predictive Control
CCS-MPC: Continuous Control Set Model Predictive Control
ERL: Exponential Reaching Law
PRRL: Power Rate Reach Law
IERL: Improved Exponential Reaching Law
ASIERL: Augmented State Improved Exponential Reaching Law
AIERL: Adaptive Improved Exponential Reaching Law
SQP: Sequential Quadratic Programming
GD: Gradient Descent
